# Supplementary material for: Neospora caninum as delivery vehicle for anti-PD-L1 scFv-Fc: A novel approach for cancer immunotherapy
Source: Mol Ther Oncol. 2025 Mar 19;33(2):200968. doi: 10.1016/j.omton.2025.200968 (PMC11999461; doi:10.1016/j.omton.2025.200968)
Supplement: Document S1. Figures S1–S6 [file mmc1.pdf]

OMTON, Volume *33*

Supplemental information

Neospora caninum as delivery vehicle for anti-PD-L1 scFv-Fc: A novel approach for cancer immunotherapy

Clément Riviere, Muna Aljieli, Marie-Noëlle Mévélec, Louis Lantier, Fanny Boursin, Laurie Lajoie, Céline Ducournau, Stéphanie Germon, Nathalie Moiré, Isabelle Dimier-Poisson, Nicolas Aubrey, and Anne di Tommaso

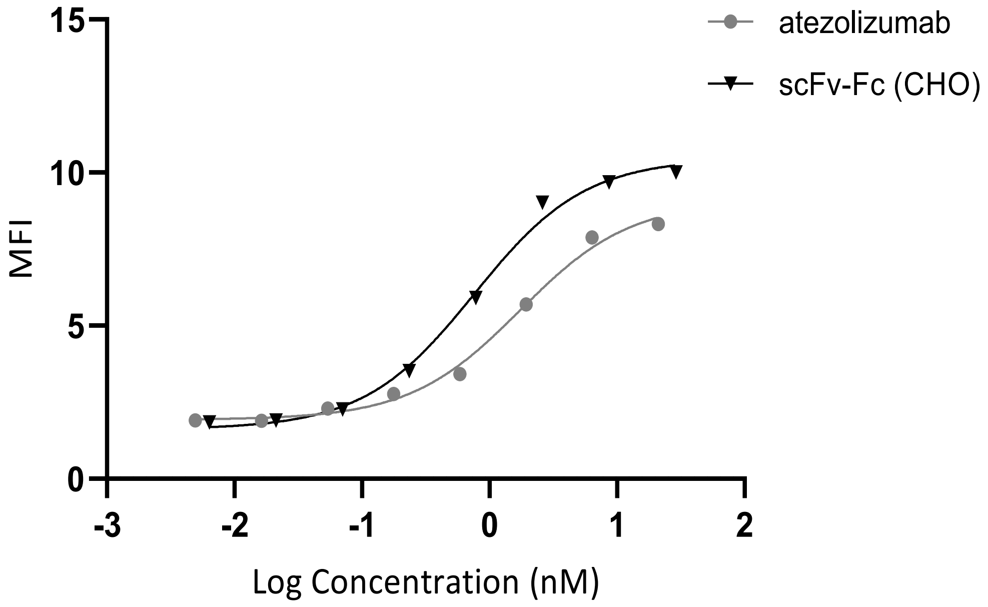

Figure S1: Anti-PD-L1 in scFv-Fc format binds to PD-L1+ tumor cells. Human tumor MDA-MB-231 cells were incubated with various concentrations of scFv-Fc purified from transfected CHO cells (scFv-Fc (CHO)) or atezolizumab (atezolizumab). MFI: Mean Fluorescent Intensity.

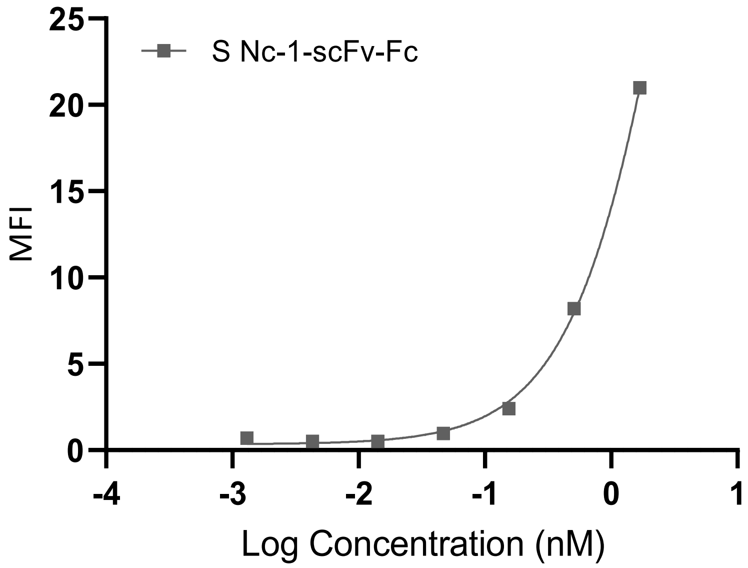

Figure S2: scFv-Fc produced by Nc-1-scFv-Fc binds to murine tumor cells. Mouse tumor cells B16F10 were incubated with various concentrations of secreted scFv-Fc corresponding to several dilutions culture supernatant of Nc-1-scFv-Fc (S Nc-1-scFv-Fc). MFI: Mean Fluorescent Intensity.

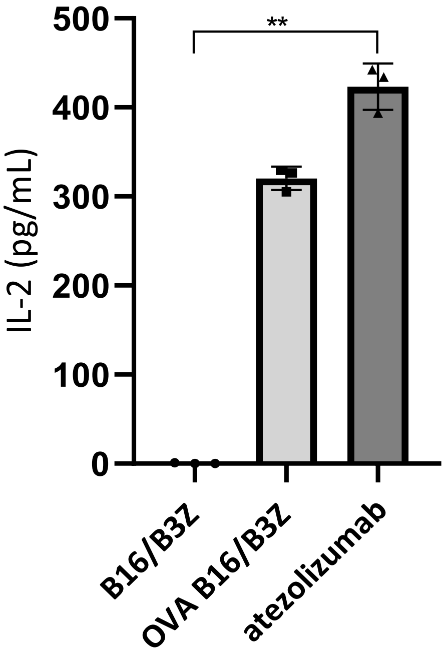

Figure S3: PD-1/PD-L1 axis limits secretion of IL-2 by B3Z cells. B16F10 cells overexpressing PD-L1 were pulsed with OVA peptide and then co-incubated with B3Z T cells (OVA B16/B3Z), in culture medium supplemented with atezolizumab (atezolizumab). As control, B16F10 cells without preincubation with OVA peptide (B16/B3Z) were co-incubated with B3Z T cells in culture medium. IL-2 production was measured by ELISA. Data are presented as mean values (n=3) and significant differences are indicated as $**p < .01$

A

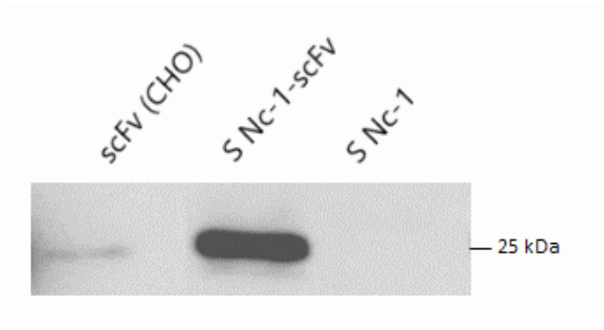

B

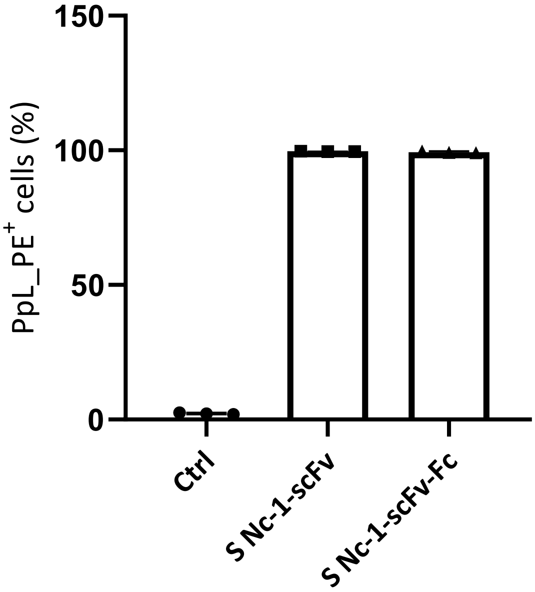

Figure S4: scFv from recombinant *Neospora caninum* binds to tumor cells. (A) Western blot was performed with culture supernatant of Nc-1-scFv (S Nc-1-scFv), culture supernatant of wild type strain (S Nc-1) and purified anti-PD-L1 scFv from CHO (scFv (CHO)). (B) Mouse tumor cells B16F10 were incubated with culture media of B16F10 (Ctrl), culture supernatant of Nc-1-scFv-Fc (S Nc-1-scFv-Fc) or culture supernatant of recombinant Nc-1-scFv (S Nc-1-scFv). Cells were analyzed by flow cytometry.

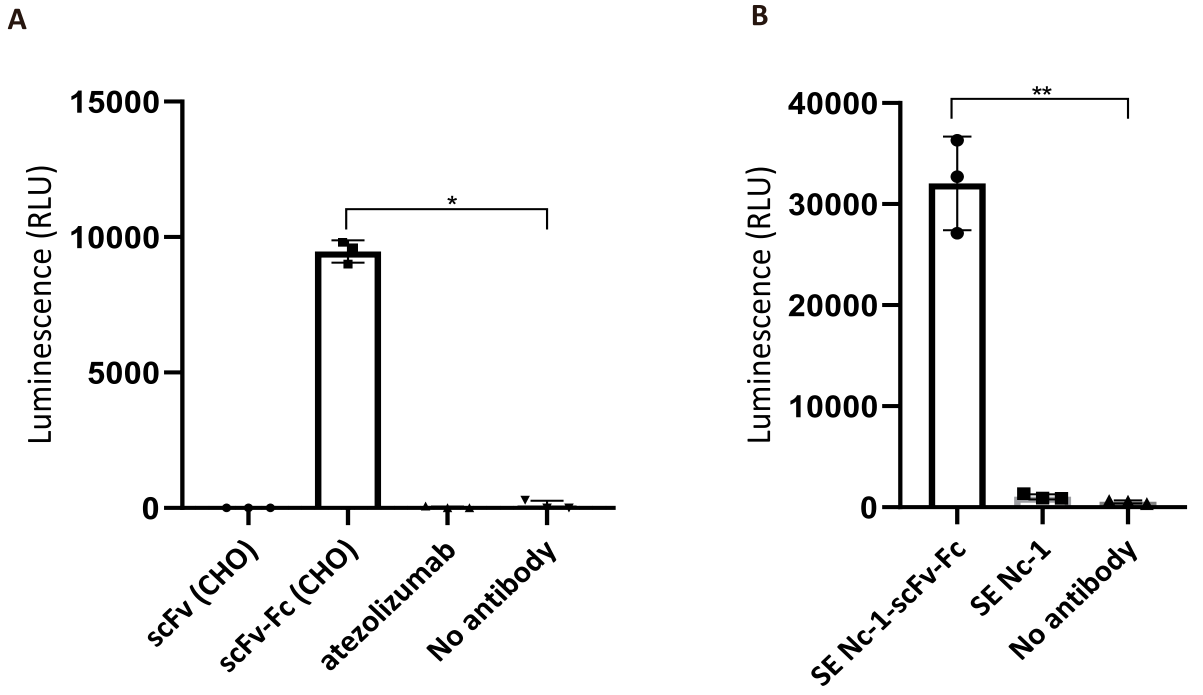

Figure S5: In vitro ADCC assay for antibody fragments produced by recombinant *N. caninum* or CHO. (A) ADCC Reporter Bioassay was made on MDA-MB-231. Target MDA-MB-231 cells were incubated with PBS (No antibody),atezolizumab (atezolizumab), or anti-PD-L1 in scFv format from CHO (scFv (CHO)) or anti-PD-L1 in scFv-Fc format from CHO (scFv-fc (CHO)) and followed, for each condition, by addition of effector cells. Luminescence was measured in relative light units. (B) In vitro ADCC assay with PBS (No antibody), enriched culture supernatant of wild type strain (SE Nc-1), enriched culture supernatant of Nc-1-scFv-Fc (SE Nc-1-scFv-Fc). Data are presented as means values (n=3) and significant differences are indicated as *$p < .05$; **$p < .01$.

A

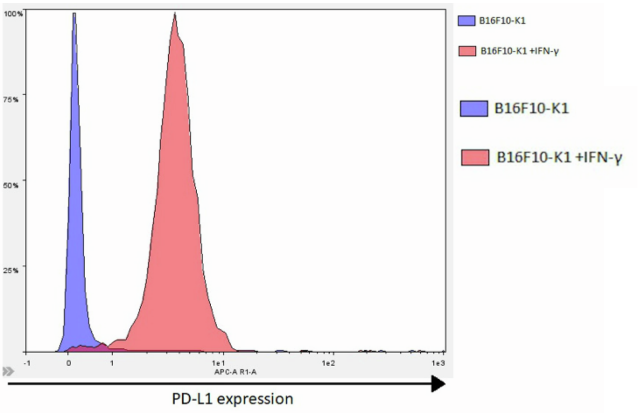

B

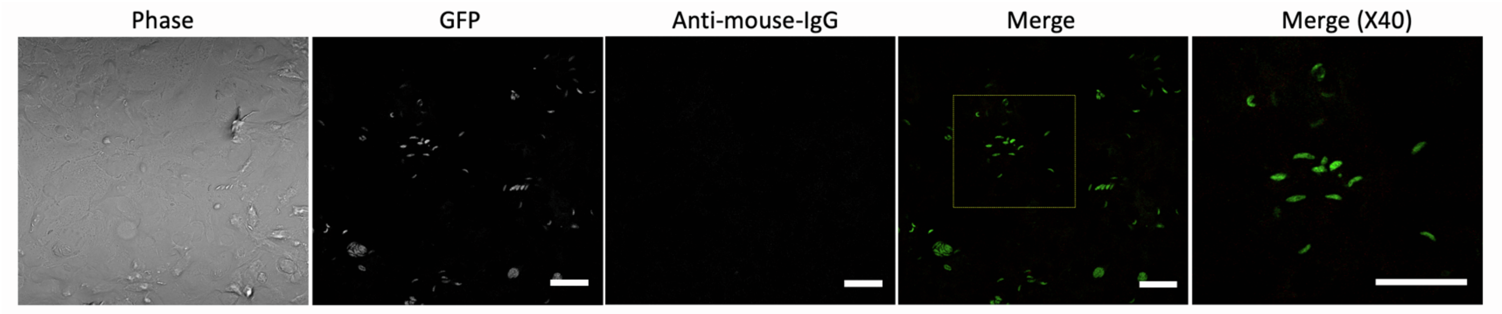

Figure S6: Binding of secreted scFv-Fc on infected or uninfected rested (non-stimulated) B16F10-K1. (A) Surface display level of PD-L1 on IFN-γ-stimulated B16F10-K1 (B16F10-K1 + IFN-γ) and non-stimulated B16F10-K1 (B16F10-K1) cells. Cells were stained with APC-conjugated mouse anti-PD-L1 before flow cytometry analysis. Results are represented as flow cytometer histograms. Profile of cells stained with anti-PD-L1 are indicated in blue area (non-stimulated B16F10-K1) and red area (IFN-γ-stimulated B16F10-K1). (B) Binding of scFv-Fc on uninfected or infected rested (non-stimulated) B16F10-K1 cells by immunofluorescence microscopy. Non-stimulated B16F10-K1 cells were incubated with Nc-1-scFv-Fc. Secreted scFv-Fc was detected with APC-conjugated anti-murine IgG (red) and individual extracellular and intracellular Nc-1-scFv-Fc were determined according to GFP expression (green). Representative results from 1 of 3 independent experiments are shown. Scale bar= 30 µm.
