# Supplementary material for: Neospora caninum as delivery vehicle for anti-PD-L1 scFv-Fc: A novel approach for cancer immunotherapy
Source: Mol Ther Oncol. 2025 Mar 19;33(2):200968. doi: 10.1016/j.omton.2025.200968 (PMC11999461; doi:10.1016/j.omton.2025.200968)
Supplement: Document S2. Article plus supplemental information [file mmc2.pdf]

Molecular Therapy
Oncology
Original Article

Neospora caninum as delivery vehicle for anti-PD-L1 scFv-Fc: A novel approach for cancer immunotherapy

Clément Riviere,[1] Muna Aljieli,[1,2] Marie-Noëlle Mévélec,[1] Louis Lantier,[1] Fanny Boursin,[1] Laurie Lajoie,[1] Céline Ducournau,[1] Stéphanie Germon,[1] Nathalie Moiré,[1] Isabelle Dimier-Poisson,[1] Nicolas Aubrey,[1] and Anne di Tommaso[1]

[1]BioMAP, UMR ISP 1282 INRAe – Université de Tours, 37200 Tours, France; [2]Faculty of Pharmacy, University of Gezira, Wad Madani, Sudan

***Neospora caninum*, a potential anticancer agent able to reactivate the immune response within the tumor microenvironment (TME), has recently shown enhanced immunomodulatory properties in different tumor models when armed with the cytokine, Il-15. In the current area of combination immunotherapy strategies designed to overcome treatment resistance, we engineered for the first time the protozoan *Neospora caninum* to vectorize and secrete a single-chain variable fragment fused to fragment crystallizable region (scFv-Fc) targeting human programmed cell death ligand 1 (PD-L1). Following validation of its secretion through the micronemes (protozoa secretory organelles), we demonstrated that the scFv-Fc could bind PD-L1 on mouse and human tumor cells, block the programmed cell death protein 1 (PD-1)/PD-L1 pathway leading to potentiate the T cell lymphocyte activity. Additionally, the scFv-Fc induced antibody-dependent cellular phagocytosis (ADCP) and antibody-dependent cellular cytotoxicity (ADCC). Those data demonstrate the feasibility of vectoring and secreting a functional antibody fragment by *N. caninum,* opening promising avenues for future research.**

INTRODUCTION

In the last decade, immunotherapy has become a critical pillar of cancer treatment.[1] Several approaches have been developed, among which immune checkpoint inhibitors (ICIs) represent a breakthrough, now clinically used in about 20 tumor types, either as monotherapy or in combination.[2] PD-1, predominantly expressed on activated T cells, induces an inhibitory signal after binding to PD-L1, overexpressed by several tumor types and therefore playing a crucial role in tumor escape. Currently, three anti-PD-L1 monoclonal antibodies (mAbs) have been approved by the Food and Drug Administration (FDA): atezolizumab (Tecentriq, 2016), durvalumab (Imfinzi, 2017), and avelumab (Bavencio, 2017). The FDA has since expanded their indications, reflecting an increase in their usage across a broader range of cancer types and disease stages, underscoring their significative as active immunotherapies.[3] However, the success of those therapies is highly dependent on the immunological status of the tumor microenvironment (TME). Tumors with an immunosuppressive microenvironment present a range of barriers to their effectiveness. Factors such as limited immune cell infiltration, the presence of suppressive molecules (e.g., interleukin [IL]-6, IL-10, transforming growth factor [TGF]β) and cells (e.g., myeloid-derived suppressor cells [MDSCs], tumor-associated macrophages [TAMs], and regulatory T cells [Tregs]), and metabolic stress (e.g., hypoxia, nutrient depletion) hinder the immune response. These conditions impair antigen presentation, promote immune exhaustion, and weaken the involvement of the PD-1/PD-L1 axis, ultimately reducing the efficacy of ICI therapy.[4–7] Tissue penetration is another challenge, particularly for solid tumors. The large size of antibodies often hinders their ability to reach the core of the tumor.[8] Overcoming these limitations represents a significant challenge and requires the development of innovative strategies. Overcoming these limitations brings the need of exploring new therapeutic strategies. Oncolytic viruses (OVs)[9] and bacteria[10] have emerged as promising therapeutic approaches. IMLYGIC (talimogene laherparepvec or T-VEC), the most notable viral therapy for advanced melanoma, demonstrates a 25% response rate.[11] Preclinical studies suggest that combining OVs with ICIs, such as anti-CTLA4 or anti-PD-1, can significantly improve antitumor responses compared with monotherapy. However, long-term survival and autoimmune-type toxicity require further improvement.[12–14] Using OV as vector for therapeutic molecules delivery, such as anti-PD-L1, holds promise, potentially reducing systemic exposure and increasing efficacy.[15] Protozoa also represent promising immunotherapeutic agents. Clinical evaluations of *Plasmodium* species and preclinical studies with *Trypanosoma cruzi* or *Toxoplasma gondii* for advanced cancers have also been investigated.[16–18] In a recent study, we demonstrated that a replicative *T. gondii* engineered to display surface anti-PD-L1 antibody fragments induces specific tumor oncolysis.[19] Moreover, combining *T. gondii* strains with an anti-PD-L1 antibody in cancer models has shown promise reducing

Received 29 July 2024; accepted 16 March 2025;
https://doi.org/10.1016/j.omton.2025.200968.

Correspondence: Anne di Tommaso, BioMAP, UMR ISP 1282 INRAe – Université de Tours, 37200 Tours, France.
E-mail: anne.ditommaso@univ-tours.fr

Published by Elsevier Inc. on behalf of The American Society of Gene and Cell Therapy.
This is an open access article under the CC BY-NC-ND license (http://creativecommons.org/licenses/by-nc-nd/4.0/).

tumor growth and increasing T cell infiltration, even in an immunosuppressive TME.[20,21]

Neospora caninum, an obligate intracellular protozoan closely related to *T. gondii*,[22] holds similar promise as an immunotherapeutic agent. Our previous work demonstrated that the Nc-1 strain of *N. caninum* inhibited murine thymoma development and impaired murine lung metastasis progression following intratumorally, local, or distant administration. This antitumoral effect is driven by a direct oncolytic activity, induction of an immune response in the TME and shifting tolerant immune cells to an immune-active state.[23–25] Moreover, we demonstrated *N. caninum* ability to secrete large protein in the TME by engineering an IL-15 secreting strain (NC-Il15hRec), which displayed enhanced anti-tumoral responses and efficacy.[23,24] Based on the relevance of combining therapeutic antibodies[20,21] and building upon our findings, the present study focuses on engineering a recombinant *N. caninum* strain to produce and secrete a therapeutic antibody against PD-L1, in a scFv-Fc format. *In vitro* experiments conducted during the infection phases of recombinant *N. caninum* demonstrated the continuous release of anti-PD-L1 scFv-Fc, which effectively bound to tumor cells including those distant from the initially infected sites. Additionally, the scFv-Fc restored lymphocyte activity by blocking the PD-1/PD-L1 pathway, enhanced ADCP, and potentially increased ADCC. Those data provide a proof of concept of antibody fragment secretion feasibility by *N. caninum* and offer a promising approach for cancer therapy.

RESULTS

Generation of a recombinant *N. caninum* strain and characterization of the secreted anti-PD-L1 scFv-Fc

The coding gene for scFv-Fc directed against PD-L1 was constructed in a VH-linker-VL orientation according to the sequence of the human monoclonal antibody atezolizumab. This was fused with the hinge region and the fragment crystallizable region of mouse immunoglobulin (Ig)G2a. To target the scFv-Fc to the microneme secretory pathway, a sequence encoding the N-terminal signal peptide and propeptide domain of *T. gondii* MIC5 was incorporated (Figure 1A). Similar to *T. gondii*, *N. caninum* possesses specialized apical secretory organelles called micronemes, which are essential for the parasite survival and dissemination. During exocytosis, micronemes release a variety of proteins, including adhesin complexes, perforins, and proteases. These proteins play critical roles in the parasite life cycle, facilitating the exit from infected cells, enabling gliding motility, crossing biological barriers, and successful invasion of host cells.[26] This construct was inserted into the plasmid pUC5 then transfected into wild-type Nc-1 to generate the Nc-1-scFv-Fc, selected by GFP expression as previously described for *T. gondii*.[27] The modified strain resulted in the secretion of recombinant scFv-Fc of approximately 110 kDa under non-reducing conditions as a disulfide-linked homodimer, as observed with the secretion of the same fragment by the reference eukaryotic expression system Chinese hamster ovary (CHO) (Figure 1B). Intracellular immunofluorescence staining with an anti-mouse IgG allowed detection of the scFv-Fc at the apical pole of intracellular Nc-1-scFv-Fc strain (blue color). This subcellular localization was similar to the endogenous microneme protein MIC3 (red color) (Figure 1C). The absence of detectable signal in the cytoplasm of tachyzoites (infectious form of *N. caninum*) or within the parasitophorous vacuole suggests that alternative secretion pathways utilized by the protozoan are not involved in the release of the recombinant protein. To further characterize the secretion of the fragment, a time course of secreted scFv-Fc in culture supernatant from freshly egressed Nc-1-scFv-Fc was investigated at 37°C, during 60 min without host cells. During this extracellular phase, intracellularly stored microneme proteins, including scFv-Fc, continue to be secreted.[28] The kinetics of scFv-Fc accumulation showed a secretion peak of 3.32 ng/mL in the first hour, highlighting the secretion dynamics in this experimental setup. Although accumulation continued throughout the 60-min sampling period, most of the scFv-Fc was secreted during the early stages following egression, with 50% of this concentration reached within 4 min (Figure 1D). The influence of temperature on the microneme secretory pathway was also investigated at temperatures ranging from 0°C to 37°C.[29] The secretion of scFv-Fc into the culture supernatant increased significantly at 29°C and 37°C ($p < 0.05$) compared with the residual secretion observed at lower temperatures (7%) (Figure 1E). To complete this secretion study, microneme discharge from freshly egressed Nc-1-scFv-Fc was induced with ethanol, previously identified as a potent stimulator for extracellular secretion of microneme proteins.[29,30] As shown in Figure 1F, total discharge of the micronemes allowed a significant scFv-Fc release of 150% ($p < 0.01$) compared with a condition without ethanol. Therefore, microneme secretion of scFv-Fc was validated through co-localization with MIC3, secretion kinetics, and ethanol-induced discharge. The absence of cytoplasmic retention further supports the specificity of the microneme pathway.

Comparable fitness and lytic activity of the Nc-1-scFv-Fc strain with the wild-type strain Nc-1

Invasion, a conserved process within the apicomplexan phylum, relies on the timely and spatially controlled sequential events throughout the lytic cycle: exocytosis, adhesion, and finally invasion of host cell.[31] During this process, microneme proteins are involved in the disruption of cell membranes for egress, as well as in the recognition and attachment to host cells,[32–34] and the impact of the scFv-Fc transgene on the strain infectivity was studied. An invasion assay was performed using human foreskin fibroblast (HFF) cells to compare the attachment and invasion capacities of Nc-1-scFv-Fc with the reference strain Nc-1. As observed in Figure 2A, attachment was observed by extracellular staining (yellow color) while invasion was characterized by intracellular staining (green color). After 30 min and 3 h following HFF infection, the total number of attached and intracellular Nc-1-scFv-Fc per 100 cells was not significantly different from Nc-1 (Figure 2B). Interestingly, the percentage of intracellular tachyzoites at each time point was similar for both strains (Figure 2C) suggesting that the scFv-Fc expression did not affect the infectivity of the Nc-1-scFv-Fc strain. In the same way, the replication of Nc-1-scFv-Fc was also similar to that of the wild type, as no significant difference in number of egressed tachyzoites was observed over later time points using murine melanoma

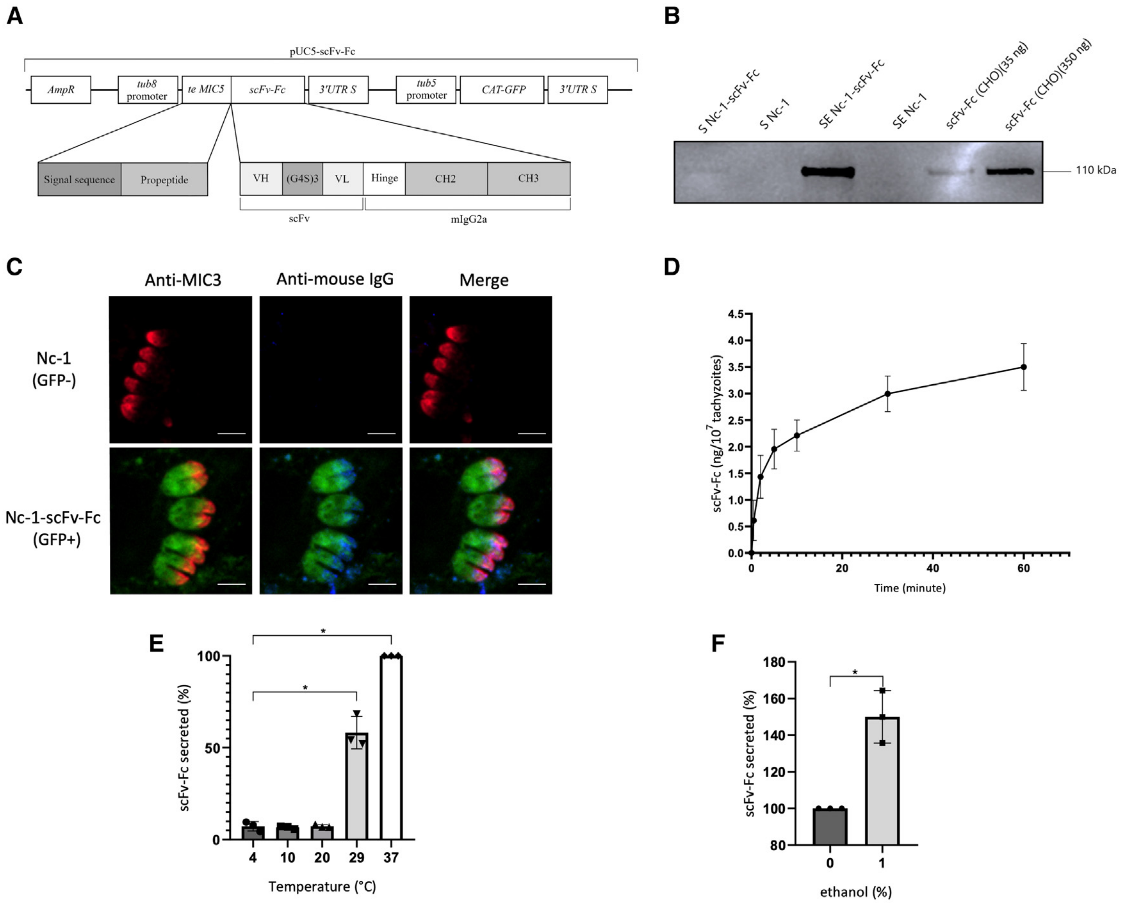

Figure 1. Generation of a recombinant *N. caninum* secreting scFv-Fc

(A) Schematic representation of pUC5-scFv-Fc vector used for the obtention of recombinant Nc-1-scFv-Fc strain. Genetic map of pUC5 plasmid including the sequence encoding anti-PD-L1 scFv-Fc fused with targeting elements of MIC5 (te MIC5) comprising the signal sequence and propeptide of MIC5. The scFv portion composed of VH and VL and fused with CH2 and CH3 portion of murine IgG2a (mIgG2a). (B) After complete lysis of HFF cells infected with Nc-1-scFv-Fc or Nc-1 supernatants (S Nc-1-scFv-Fc and S Nc-1) and enriched supernatant (SE Nc-1-scFv-Fc and SE Nc-1) were analyzed by western blot. scFv-Fc (CHO) corresponds to purified scFv-Fc produced in CHO cells. (C) HFF cells infected with Nc-1 or Nc-1-scFv-Fc were stained with anti-MIC3 (red) and anti-mouse IgG (blue) and individual intracellular Nc-1-scFv-Fc are observed according to GFP expression (green). Overlapping of red and blue signals resulted in merge images (pink). (D) Supernatants were collected at different times and concentrations of scFv-Fc were reported in nanogram per 10^7 tachyzoites. (E and F) Temperature and ethanol influence on scFv-Fc secretion. Extracellular Nc-1-scFv-Fc were incubated at different temperatures (E) or in 1% ethanol (F). Condition at 37°C and without ethanol were respectively designated as reference (100%). For (D), (E), and (F), data are expressed as mean values ±SD ($n = 3$); significant differences are indicated as $^{*}p < 0.05$).

cells B16F10 (Figure 3A, upper). Over this time, continuous cycles of infection in B16F10 cells enabled sustained production and accumulation of scFv-Fc in the culture supernatant, reaching a concentration of 15.5 ng/mL 96 h post infection (hpi) (Figure 3A, lower). *Neospora caninum* infection of target cells is essential for its multiplication, resulting in direct oncolytic activity. Parasitophorous vacuoles were observed in B16F10 cells (Figure 3B, black arrow), and their rupture led to host cell lysis, confirming a direct oncolytic effect (Figure 3B, white arrow). The oncolytic activity of both Nc-1-scFv-Fc and Nc-1 strains did not show any significant difference at different MOIs 72 hpi. (Figure 3C). Overall, secretion of scFv-Fc does not appear to have an effect on the oncolytic capacity of the recombinant protozoa.

Anti-PD-L1 scFv-Fc secreted by *N. caninum* blocks the PD-1/PD-L1 pathway

The capacity of scFv-Fc to recognize and bind to PD-L1 expressed on the surface of human tumor cells was evaluated by flow cytometry. Compared with atezolizumab, the scFv-Fc produced by CHO cells showed similar binding affinity to human PD-L1 protein (Figure S1) with a negligible difference of half maximal effective concentration values (0.8 and 1.6 nM, respectively). Incubation of human breast cancer cell line MDA-MB-231, which expresses high level of PD-L1 on the cell membrane,[19] with culture supernatant of Nc-1-scFv-Fc led to a median fluorescence intensity of up to 7 while the positive control (scFv-Fc produced in CHO) displayed a median fluorescence intensity (MFI) of 13 (Figure 4A). Although the concentration of

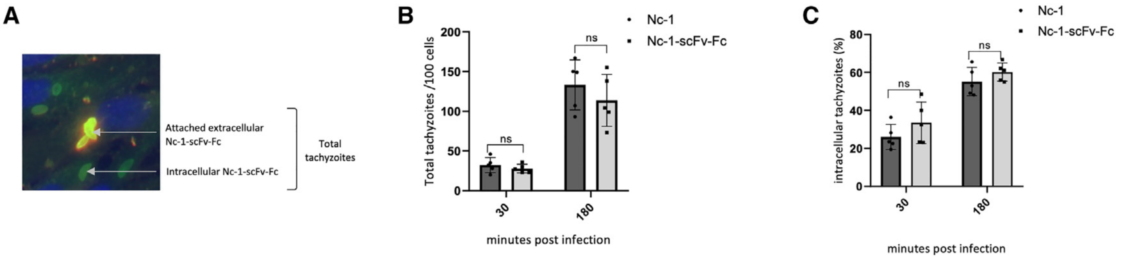

Figure 2. Infectious capacity of Nc-1-scFv-Fc evaluated in invasion assay

(A) Attached extracellular tachyzoites (yellow) and intracellular tachyzoites (green) were labeled with anti-Nc-1. Host cell nuclei were stained with Hoechst (blue). (B) Attached extracellular and intracellular tachyzoites (total) per 100 HFF cells and (C) intracellular tachyzoites indicated as percentages based on total tachyzoites. Total numbers and intracellular tachyzoites were counted for five randomly selected fields of each strain. (B) and (C) are representative of three independent experiments with similar results. ns: not significant.

scFv-Fc available in the culture supernatant was insufficient to reach the stationary phase in the binding assay, the scFv-Fc secreted by Nc-1-scFv-Fc exhibited a dose-dependent binding affinity to PD-L1 similar to scFv-Fc purified from CHO cells (used as control). Similar results were obtained with murine melanoma cell line B16F10, highlighting comparable affinity for murine PD-L1 (Figure S2). As illustrated in Figure 4B, neutralization with atezolizumab led to a concentration-dependent inhibition of scFv-Fc binding to MDA-MB-231 cells with maximal inhibition achieved at a saturating concentration of atezolizumab (1.7 nM). These findings suggest that the secreted scFv-Fc can efficiently bind to both human and murine tumor cells and this cell binding is dependent upon surface display of PD-L1.

The therapeutic effect of atezolizumab involves its capacity to inhibit the PD-1/PD-L1 pathway. To test the inhibitory effect of scFv-Fc against PD-L1, B16F10 cells were stimulated to overexpress PD-L1 (with interferon [IFN]-γ stimulation) before co-incubation with recombinant PD-1, anti-PD-L1 scFv-Fc or controls. Whereas recombinant PD-1 bound to PD-L1 expressing B16F10 cells in the presence of controls (i.e., PBS, rituximab [irrelevant antibody], culture supernatant from the Nc-1 (S Nc-1), or culture supernatant from a genetically modified *N. caninum* secreting an irrelevant scFv (S Nc-1-scFv_Irr)), this binding was significantly disrupted by 96% with culture supernatant from Nc-1-scFv-Fc (S Nc-1-Nc-scFv-Fc) ($p < 0.05$) (Figure 4C). Similar inhibition results were obtained for conditions with atezolizumab or supernatant of Nc-1 complemented with purified scFv-Fc from CHO (S Nc-1+scFv-Fc (CHO) reaching 2.1% and 2.5%, respectively ($p < 0.05$) (Figure 4C). Next, the ability of the scFv-Fc to promote T cell responses was evaluated in an *in vitro* co-culture assay. B16F10 cells incubated with MHC-I-restricted peptide SIINFEKL (OVA257-264) were used as target cells. Murine B3Z CD8+ T cells, which recognize SIINFEKL bound to H-2Kb MHC-I, were used as antigen-specific CD8+ effector T cells. B16F10 cells were stimulated to overexpress PD-L1 by IFN-γ before co-culture with B3Z in the

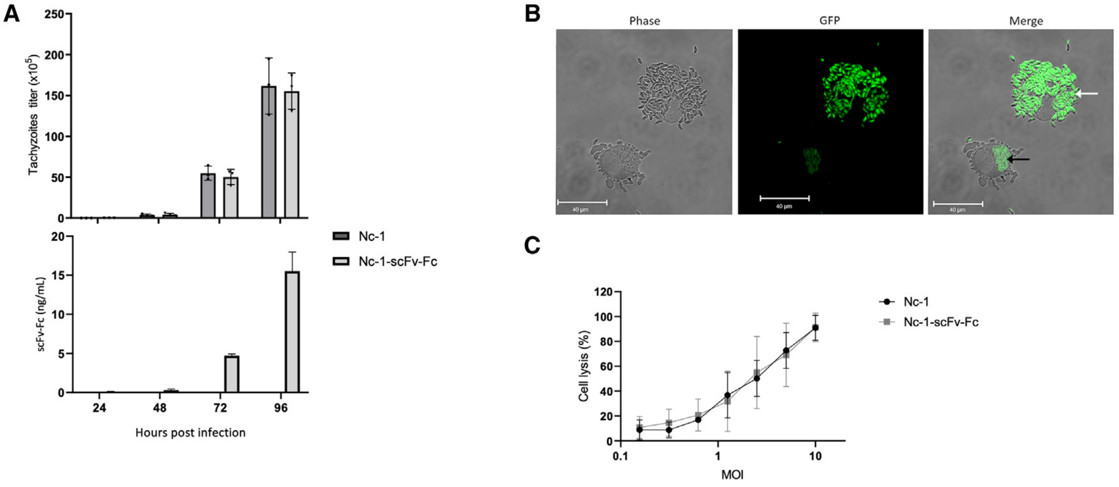

Figure 3. *In vitro* replication and oncolytic activity of Nc-1-scFv-Fc

(A) Following infection of B16F10 cells, extracellular tachyzoites were counted and the concentration of secreted scFv-Fc was measured in ng/mL. (B) Imaging of lysis of B16F10 cells infected by Nc-1-scFv-Fc 72 hpi (at ×40). Black and white arrows designate intracellular and extracellular tachyzoites, respectively. (C) Cell viability of B16F10 infected with Nc-1-scFv-Fc or Nc-1 at the indicated MOIs was measured by bioluminescence assay. Data are expressed as mean values ±SD ($n = 3$).

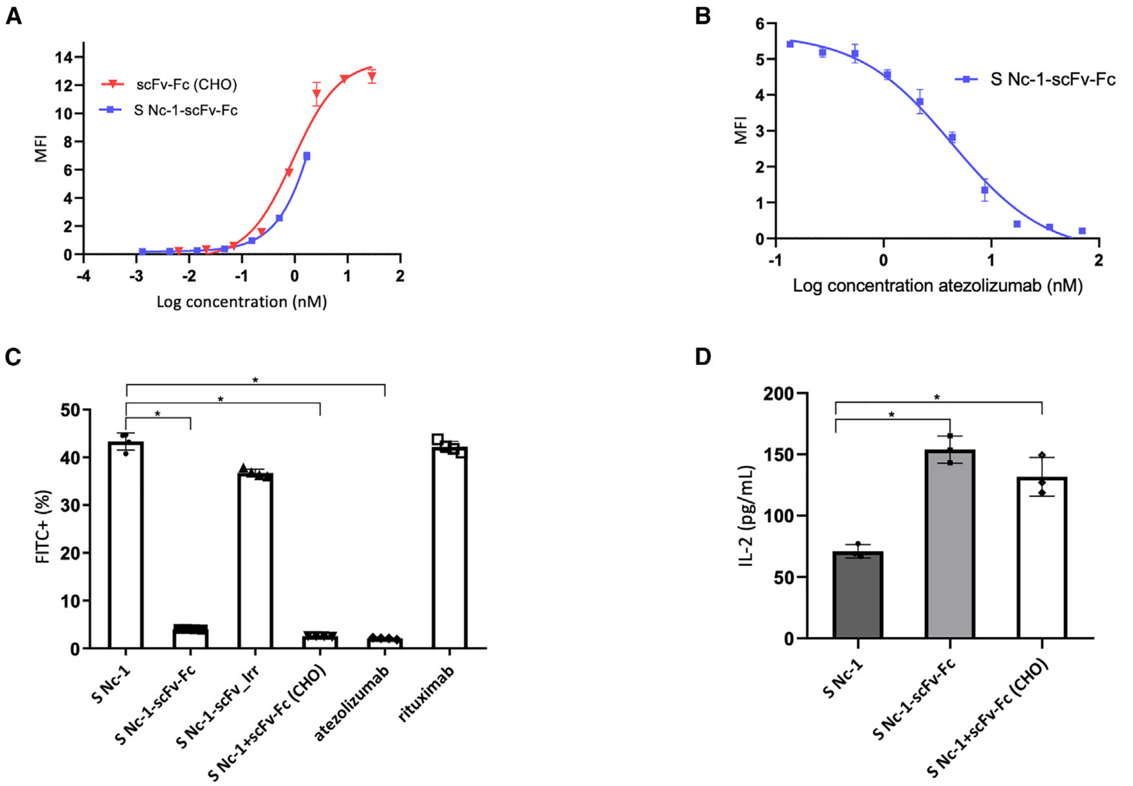

Figure 4. Binding and blocking effect of scFv-Fc on PD-L1

(A) MDA-MB-231 cells were incubated with scFv-Fc from transfected CHO cells (scFv-Fc [CHO]) or secreted by Nc-1-scFv-Fc (S Nc-1-scFv-Fc). (B) MDA-MB231 cells were incubated with increased concentrations of atezolizumab followed by incubation with culture supernatant of Nc-1-scFv-Fc (S Nc-1-scFv-Fc). (C) Binding of rPD-1 to B16F10 cells pre-incubated in medium with irrelevant antibody (rituximab), culture supernatant of Nc-1 (S Nc-1) alone or complemented with scFv-Fc purified from CHO cells (S Nc-1+scFv-Fc [CHO]), culture supernatant of Nc-1-scFv-Fc (S Nc-1-scFv-Fc), of a recombinant *N. caninum* expressing an irrelevant scFv (S Nc-1-scFv_Irr) or atezolizumab. Fixation of rPD-1 on cells was detected by flow cytometry. (D) B16F10 cells overexpressing PD-L1 pulsed with OVA peptide were co-incubated with B3Z T cells PD-1+ in culture supernatant of either Nc-1 alone (S Nc-1), supplemented with scFv-Fc from CHO cells (S Nc-1 + scFv-Fc [CHO]), or of Nc-1-scFv-Fc (S Nc-1-scFv-Fc). Concentration of IL-2 was measured by ELISA. Data are expressed as mean values ±SD (n = 3; significant differences are indicated as *$p < 0.05$). MFI, median fluorescence intensity.

presence of culture supernatant from either Nc-1 (as control), Nc-1-scFv-Fc or Nc-1 complemented with purified scFv-Fc from CHO. The specific activity of B3Z cells against B16F10 presenting OVA peptide was reported by IL-2 expression. In conditions with scFv-Fc secreted by Nc-1-scFv-Fc or purified from CHO, IL-2 secretion was significantly enhanced compared with condition with supernatant of Nc-1 reaching 153 pg/mL and 132 pg/mL, respectively (Figure 4D) ($p < 0.05$). The same result was obtained using atezolizumab (Figure S3). Altogether, these data demonstrated the capacity of the scFv-Fc secreted by Nc-1-scFv-Fc to bind PD-L1 and blocked the inhibitory effect of immune cells.

Anti-PDL-1 scFv-Fc secreted by *N. caninum* induces ADCP and ADCC

The Fc region of antibodies mediates various functions, including binding to Fc receptors on immune cells to induce ADCC or ADCP.[35] To evaluate whether scFv-Fc could induce ADCP, we first assessed its binding to murine macrophages RAW 264.7.[36] Compared with the control, only the scFv-Fc secreted by Nc-1-scFv-Fc exhibited significant binding to the RAW 264.7 cells ($p < 0.01$) (Figure 5A). An anti-PD-L1 fragment in the scFv format, which lacks the Fc portion (used as control), showed significant binding to PD-L1+ B16F10 cells (Figures S4A and S4B) but failed to bind to RAW 264.7 cells (Figure 5A). Those results highlight the crucial role of the Fc portion in macrophage binding. IFN-γ-stimulated B16F10 cells expressing mCherry fluorescent protein were mixed at a 1:1 ratio with RAW 264.7 cells stained with fluorescein isothiocyanate (FITC)-conjugated anti-murine CD11b antibody. The phagocytosis process was analyzed by flow cytometry focusing on double-positive events ($FITC^+$ $mCherry^+$). In the presence of culture supernatant from either Nc-1 or Nc-1-scFv, the double-positive cell reached 7%, which was significantly lower compared with culture supernatant from Nc-1-scFv-Fc with approximately 13% of double-positive cells ($p < 0.05$) (Figure 5B). In addition, culture supernatant of Nc-1 complemented with purified scFv-Fc from CHO demonstrated similar results to scFv-Fc ($p < 0.05$). Taken together, those results suggest that the scFv-Fc produced by the recombinant *N. caninum* induces phagocytosis as well as an antibody fragment produced in mammalian cell lines.

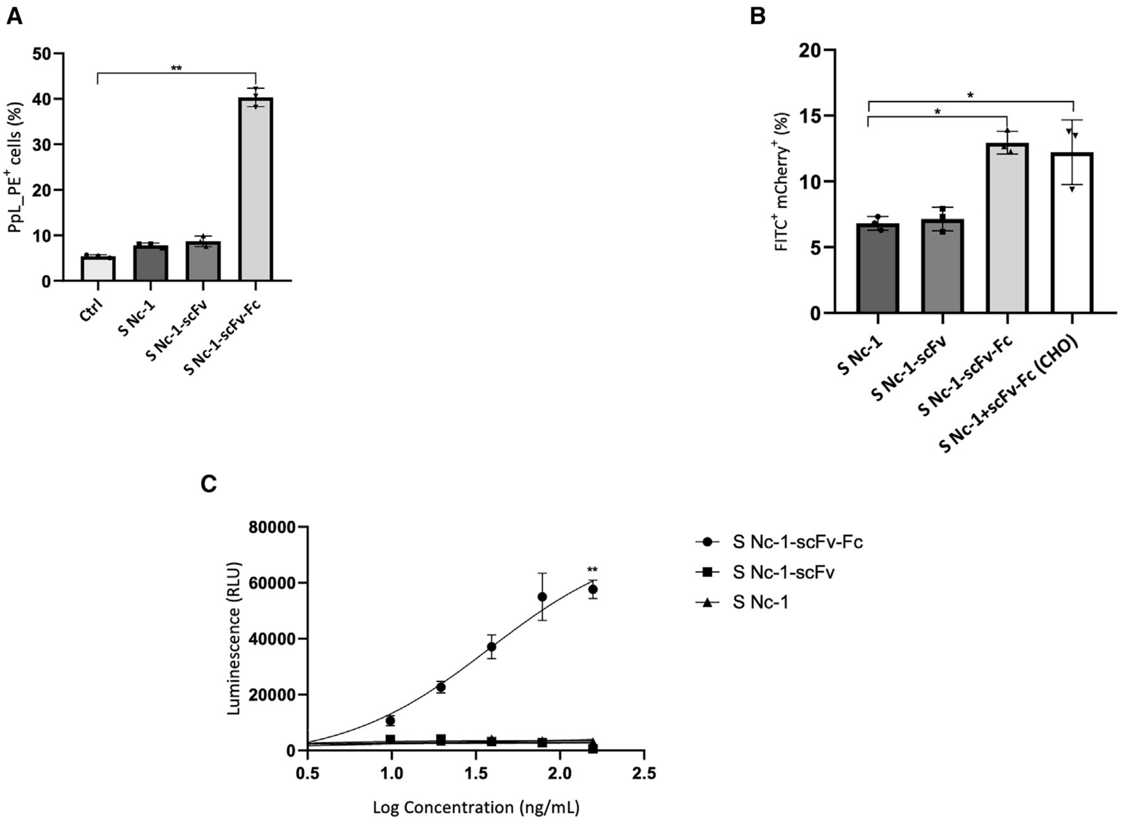

Figure 5. Induction of phagocytosis and ADCC with scFv-Fc secreted by Nc-1-scFv-Fc

(A) RAW-264.7 cells were incubated with culture supernatant from HFF cells alone (Ctrl) or infected with Nc-1 (S Nc-1), Nc-1-scFv (S Nc-1-scFv-), or Nc-1-scFv-Fc (S Nc-1-scFv-Fc). Bound antibody fragment was detected using protein L (PpL_PE). (B) B16F10 cells expressing mCherry were co-cultured with Raw-264.7 at a ratio of 1:1. Raw 264.7 in culture supernatant of Nc-1 alone (S Nc-1), S Nc-1 complemented with purified scFv-Fc from CHO cells (Nc-1+scFv-Fc [CHO]), of Nc-1-scFv (S Nc-1-scFv) or of Nc-1-scFv-Fc (S Nc-1-scFv-Fc). The level of phagocytosis was defined with double-positive events (FITC+/mCherry+). (C) ADCC Reporter Bioassay performed with culture supernatants of either Nc-1 (S Nc-1), Nc-1-scFv (S Nc-1-scFv), or Nc-1-scFv-Fc (S Nc-1-scFv-Fc). Luminescence was measured in relative light units. Data are expressed as mean values ±SD (n = 3; significant differences are indicated as $*p < 0.05$; $**p < 0.01$).

To evaluate the capacity of the scFv-Fc to bind murine FcγR and to induce ADCC, a mouse FcγRIV Reporter Bioassay with PD-L1-positive MDA-MB-231 cells was conducted. The relative light unit (RLU), which depends on the concentration of scFv-Fc secreted by the recombinant strain, reached 6.10^4 at a concentration of 79 nM. In contrast, luminescence levels for culture supernatants from Nc-1 or Nc-1-scFv were negligible, with RLUs of approximately 3.10^3. These results highlight that only scFv-Fc effectively binds to murine FcγR through the murine Fc portion and induces ADCC against PD-L1-expressing tumor cells (Figure 5D). Similar results were obtained with an RLU of 3.10^4 for the enriched supernatant of Nc-1-scFv-Fc and demonstrated a much higher signal compared with the enriched supernatant of Nc-1 or atezolizumab (Figures S5A and S5B).

Secreted anti-PD-L1 scFv-Fc binds on distant, uninfected tumor cells in a 2D cell culture model

To assess whether scFv-Fc secreted by Nc-1-scFv-Fc bound specifically in closed proximity to infected cells and/or to distant cells from the infection site, we studied the localization of scFv-Fc in a 2D cell model corresponding to monolayer cell culture. In this model, infected (GFP+) and uninfected (GFP−) MDA-MB-231 PD-L1-positive cells were distinguished by flow cytometry based on the constitutive GFP expression by the recombinant strains (Nc-1-scFv-Fc and Nc-1-scFv_irr), while scFv-Fc binding to cell surface was detected using an anti-murine IgG conjugated to APC+ (Figure 6A). As shown in Figure 6B, scFv-Fc was detected on uninfected cells (GFP− APC+). The binding of the antibody fragment to uninfected cells increased in a dose-dependent manner with the number of tachyzoites used for infection, with binding rates of 8%, 37%, and 69% at MOIs of 0.25, 0.5, and 1, respectively. Although the proportion of scFv-Fc bound to infected cells appeared to be slightly higher compared with uninfected cells, the ratio of binding to infected vs. uninfected cells followed a similar trend across all MOIs (Figure 6C). Microscopy observations of murine melanoma IFN-γ-stimulated B16F10-K1 cells infected with Nc-1-scFv-Fc (Figure 6D, upper panel) or Nc-1 (used as negative control) (Figure 6D, lower panel) showed scFv-Fc binding to both infected and adjacent uninfected cells. To assess the impact of varying PD-L1 expression levels on binding, two levels of PD-L1

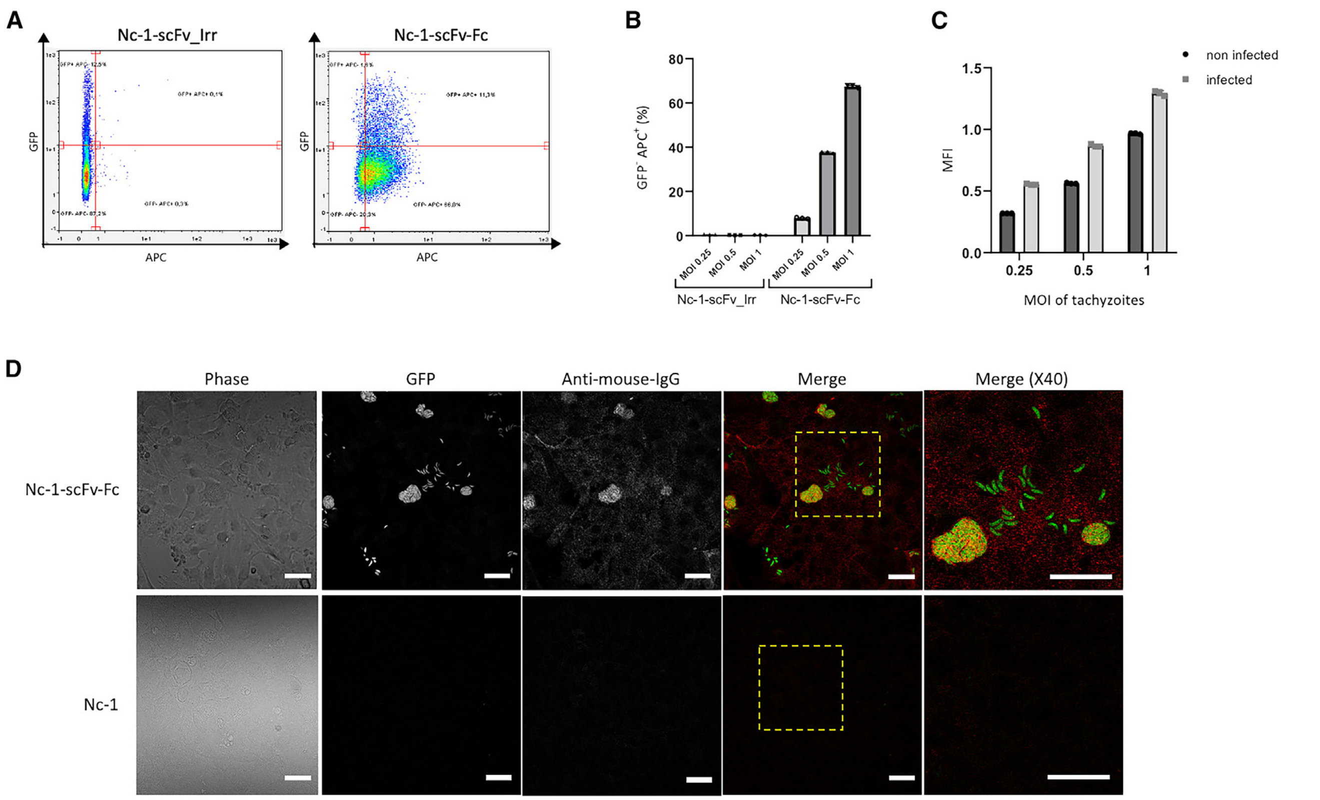

Figure 6. Binding of secreted scFv-Fc on infected or uninfected PD-L1+ tumor cells

(A) Representative flow cytometry dot blots showing fixation of scFv-Fc on uninfected cells. MDA-MB-231 cells were infected with recombinant Nc-1-scFv-Fc or Nc-1-scFv_Irr at different MOIs. ScFv-Fc was detected on uninfected cells (GFP−) or infected cells (GFP+). (B) Quantification of scFv-Fc bound to uninfected cells from experiment described in (A). Data are presented as mean values (*n* = 3). (C) Distribution of scFv-Fc between infected and uninfected cells measured by MFI. Data are presented as mean values (*n* = 3). (D) Binding of scFv-Fc on uninfected or infected B16F10-K1 cells by immunofluorescence microscopy. IFN-γ-stimulated B16F10-K1 cells were incubated with Nc-1-scFv-Fc or Nc-1. Secreted scFv-Fc was detected with APC-conjugated anti-murine IgG (red) and individual extracellular and intracellular Nc-1-scFv-Fc were determined according to GFP expression (green). Representative results from one of three independent experiments are shown. Scale bar, 30 μm. MFI, median fluorescence intensity.

expression were generated, low PD-L1 expression for rested (non-stimulated) B16F10-K1 (B16F10-K1) and high PD-L1 expression for IFN-γ-stimulated B16F10-K1 (B16F10-K1 + IFN-γ) (Figure S6A). Ligand-receptor interactions are generally influenced both by the affinity of the ligand for its receptor but also the expression level of the receptor. As expected, scFv-Fc binding followed the pattern B16F10-K1-IFN-γ > B16F10-K1, showing a strong correlation between scFv-Fc binding intensity and PD-L1 expression levels (Figure S6B). Those findings suggest that scFv-Fc secreted into the extracellular environment can effectively reach and interact with tumor cells beyond the immediate vicinity of the infected cells, with binding dependent on the surface expression of PD-L1.

DISCUSSION

Immunotherapy aims to stimulate both local and systemic immune responses, especially against cancer.[37] Although ICIs have demonstrated efficacy, clinical outcomes have often been disappointing due to severe side effects[38,39] and resistance.[40] This primary mode of resistance to ICIs is the inherently immunosuppressive nature of nonresponsive TME.[41,42] However, microbial-mediated therapies using either natural or genetically modified microorganisms have shown promise in enhancing tumor sensitivity to immunotherapy by modulating the TME.[43,44] Many viruses have been engineered as vector carriers for delivery of PD-1 or PD-L1 blockers, demonstrating improved therapeutic responses and potentially reducing systemic toxicity.[45,46] In this current study, we developed a recombinant strain of *N. caninum* that, for the first time, secretes a functional anti-PD-L1 scFv-Fc derived from atezolizumab. Previous reports have highlighted the potential of engineering protozoa to express heterologous proteins, such as *Trypanosoma cruzi* expressing human IL-2 or mouse IFN-γ.[47,48] Methods for generating stable protozoan transfectants have also been described for organisms like *T. gondii*[27,49,50] and these methods have been applied to *N. caninum*.[23] Following this approach, we generated a stable recombinant *N. caninum* strain able to secrete anti-PD-L1 scFv-Fc. The fusion of scFv with Fc is a well-established strategy to enhance bioactivity through homodimerization.[51] Notably, the secretion of two antibody fragments (scFv and scFv-Fc) has been already demonstrated in the unicellular eukaryote,

Leishmania tarentolae, as production system.[52,53] In our model, the MIC5-derived signal and propeptide sequences[54] played a pivotal role in directing scFv-Fc to the specialized apical secretory organelles of *N. caninum*. This ensured the antibody fragment was correctly secreted during the extracellular phase of the protozoan life cycle. In contrast, the CAT-GFP fusion protein lacking the MIC5 sequence remained confined to the cytoplasm. These findings, coupled with previous studies, highlight the necessity of the MIC5 propeptide for proper protein folding, trafficking, and secretion.[19,54] Such design considerations are critical for optimizing the secretion of heterologous proteins in apicomplexan parasites. Proteolytic processing of the targeting signal did not appear in the mature protein as indicated by a single band at the expected size of 110 kDa for the scFv-Fc bivalent structure. Furthermore, the similar location of scFv-Fc with the microneme protein MIC3 and the influence of ethanol on its secretion confirmed that the antibody fragment followed the microneme secretion pathway. Consistent with microneme protein secretion, scFc-Fc secretion remained active throughout the extracellular phase of the protozoan with significant protein release within the first 10 min.[30] While *N. caninum* can invade and multiply within any nucleated cell,[55] scFv-Fc production was detected in the culture supernatant of both infected tumor and non-tumor cells, without determining if the type of infected cells influenced production levels. In our model, scFv-Fc production was in the nanogram range per 1.10^7 tachyzoites within the first hour after egress, consistent with the production of interleukin (IL)-15 by our previous recombinant *N. caninum* strain (e.g., C.R. and A.d.T, unpublished data), IFN-γ produced by *T. gondii,*[50] or the scFv-Fc secreted by *L. tarentolae*.[52] Although IL-15 levels required for antitumor activity in mouse models were in the range of 2–3 μg,[56,57] the quantities used in preclinical studies for effective antitumor response with anti-PD-L1 were much higher.[20,21] Systemic injections typically require large amounts of antibody to overcome limited penetration and reduce systemic side effects.[58] Intratumoral delivery of therapeutic molecules, such as antibodies, has been proposed as an alternative strategy to minimize off-target toxicities and reduce drug dosages.[59–61] Given this, the amount of scFv-Fc produced by the recombinant *N. caninum* raises questions about its biological effect, even with intratumoral delivery. Thus, it was essential to evaluate the functionality of the scFv-Fc and to determine if the secreted amount could generate a potential antitumor activity. We first demonstrated that scFv-Fc binds to human and murine PD-L1 on tumor cell surfaces blocking interactions with PD-1 and limiting immune checkpoint inhibition. The direct lytic effect of *N. caninum*, combined with the activation of innate immune cells primed for adaptive immune responses against exogenous and tumor-associated antigens, was previously reported with the OVA peptide in the EG7 model.[23] In our co-culture system, scFv-Fc enhanced T cell lymphocyte activity against the specific antigen, resulting in a 2-fold increase in IL-2 secretion. Additionally, like scFv-Fc from CHO cells, which is known for functional Fc-mediated ADCC and ADCP,[62] the Fc portion of scFv-Fc produced by *N. caninum* significantly induced ADCP and ADCC. Recent studies emphasized the central role of N-linked glycans in FcγR-mediated effector functions.[63] While N-glycosylation has been well documented in only two Apicomplexa species, *Plasmodium falciparum* and *T. gondii*,[64,65] our results are the first to argue that *N. caninum* can certainly promote sufficient N-glycosylation required for functional cytotoxicity. It is known in literature that the Fc region of atezolizumab has been engineered to reduce Fc effector functions to minimize depletion of PD-L1-expressing effector cells and associated side effects (caused by the induction of ADCC or ADCP).[66] The relevance of a functional Fc in the therapeutic activity of PD-L1-targeting antibodies remains debated.[67] Evaluating the added value of the cytotoxic effects induced by the secreted scFv-Fc from *N. caninum* could be insightful. Similar to the strain secreting IL-15,[23,24] scFv-Fc was vectorized and delivered locally, with primarily detection near the secreting tachyzoites (e.g., C.R. and A.d.T, unpublished data). To better assess the binding dynamics of scFv-Fc to tumor cells and its spatial distribution after infection with *N. caninum*, further studies exploring tissue clearing 3D tumor cell models will be carried out. Those advanced spheroid models aim to partially replicate the structural complexity of native tissues incorporating extracellular matrix (ECM) components to more accurately reflect the tumor environment. Additionally, these studies will assess the impact of recombinant *N. caninum* infection on tumor architecture and scFv-Fc-mediated immune responses. The results of these studies are expected to provide valuable parameters for refining this approach and enhancing its potential clinical applications, especially in advancing vectorized immunotherapy using microorganisms like *N. caninum*. This strategy holds great promise for delivering biotherapeutics to hard-to-reach tumors while effectively addressing mechanisms of tumor resistance. While *N. caninum* shows promising potential as an innovative vector for antitumor immunotherapy, its biological safety profile requires thorough evaluation. Current evidence, including our recent studies[23,24] and supporting literature,[68,69] highlights the low zoonotic risk associated with *N. caninum* and its inability to establish infection in humans. However, further investigations would be necessary to rigorously define its safety, particularly for clinical applications.[70] Addressing these parameters will not only ensure safety but also facilitate the transition of this promising therapeutic strategy to clinical trials and eventual patient use. In conclusion, the production of functional antibody derivatives by microorganisms like *N. caninum* represents a promising approach to enhance antitumor efficacy and opens the door to various combination therapies.

MATERIALS AND METHODS

Protozoa

Neospora caninum (Nc-1) obtained from ATCC (50843) were routinely grown at 37°C in human foreskin fibroblasts (HFFs) as described previously.[71]

Generation of recombinant *N. caninum* strains

A synthetic sequence (GenScript) encoding the scFv anti-PD-L1 was generated by assembling the heavy (VH) and light (VL) variable domains of atezolizumab (9814_H and 9814_L, respectively; ImMunoGene Tics [IMGT] access no. 9814) linked with the peptide linker $(Gly_4Ser)_3$ (synthesized by GenScript). For targeting to micronemes, the sequence of VH was linked to the N-terminal sequence

and cleavable prodomain motif of the *Toxoplasma gondii* MIC5 protein.[54] The mouse Fc (IGHG2A*01; IMGT access no. V00825) generated by GenScript was amplified by PCR using two primers 5′-CCAAAGTGGAGATTAAAgaacctcgaggacccactatcaagcc-3′ and 5′-GCGGCCGCTTActtccctgg-3′ before insertion at the 3′ end of the VL domain by Overlap-Extension PCR to obtain the anti-PD-L1 scFv-Fc sequence. All these sequences were optimized for the protozoan organism (https://eu.idtdna.com/CodonOpt). The sequence of scFv-Fc was cloned into the previously described plasmid pUC5[19] using *Pme*I and *Not*I restriction sites (New England Biolabs) to obtain pUC5-scFv-Fc. The sequences for all newly constructed genes and plasmids were confirmed by DNA sequencing (GATC Online). *N. caninum* strain Nc-1 tachyzoites were electroporated with pUC5-scFv-Fc to obtain Nc-1-scFv-Fc strain. Transfectants were selected with 20 μM of chloramphenicol before cloning by limiting dilution.[19]

To obtained recombinants Nc-1-scFv and Nc-1-scFv_Irr, similar protocol was applied. Synthetic sequences encoding the scFv anti-PD-L1 and scFv_Irr were generated by assembling the VH and VL variable domains of atezolizumab and monalizumab, respectively (synthesized by GenScript).

Production of anti-PD-L1 in scFv and scFv-Fc formats by CHO cells

scFv-Fc and scFv were produced by CHO cells with the same amino acid sequence as the scFv-Fc produced by *N. caninum* following the protocol already described.[72] The supernatant was harvested and purified with an Akta purifier using a HiScreen Capto L column (Cytiva Europe GmbH) and scFv-Fc was eluted by a linear pH gradient in 0.1 M glycine buffer running from pH 6 to pH 2, and the buffer was removed by a desalting column. Antibody fragment concentrations were determined at 280 nm, and their theoretical molecular mass and molar coefficients of extinction were defined using protparam tool of Expasy, as follows: for scFv, 249 amino acids, 26510.3 Da, and $\varepsilon = 2417/\text{mg}\cdot\text{mL}\cdot\text{cm}$; and for scFv-Fc, 473 amino acids, 51936,5 Da, and $\varepsilon = 1779/\text{mg}\cdot\text{mL}\cdot\text{cm}$. The size and integrity of purified scFv and scFv-Fc were assessed by sodium dodecyl sulfate-polyacrylamide gel electrophoresis (SDS-PAGE) on homogeneous 12% polyacrylamide gel, under denaturation and reducing or non-reducing conditions. Purified scFv samples were all loaded at 1 μg for Coomassie Blue staining (0.1% Coomassie Brilliant Blue R-250, 30% methanol, and 10% glacial acetic acid).

Cell lines and cell culture

Murine melanoma cell lines B16F10 expressing mCherry and firefly luciferase and B16F10-K1 were cultured in RPMI 1640 medium with 10% fetal bovine serum (FBS), penicillin, and streptomycin. The human breast cancer cell line MDA-MB-231 was cultured in the same medium with 1% non-essential amino acid (NEAA). Murine monocyte-macrophage cell line RAW 264.7 and HFF cells were cultured in high glucose DMEM with 10% FBS and antibiotics. Murine T cell hybridoma cell line B3Z was grown in RPMI with supplements. All cells were cultured at 37°C in 5% CO_2. For all flow cytometry and immunofluorescence experiments, B16F10 and B16F10-K1 cell lines, whose basal PD-L1 expression is moderate to low respectively,[19] were treated with 10 ng/mL of recombinant murine interferon gamma (IFN-γ Gibco, Thermo Fisher Scientific) for 24 h to up-regulate PD-L1 expression. PD-L1 overexpression on B16F10 and B16F10-K1 cells as well as rested (non-stimulated) B16F10-K1 cells was analyzed by staining 5.10^5 cells with APC-conjugated murine anti-PD-L1 (Invitrogen) for 30 min on ice. The fluorescence intensity of the cells was determined by flow cytometry (MACS Quant, Miltenyi Biotec), and data were analyzed using FlowLogic software Version 7.2.1 (Miltenyi Biotec).

Production of scFv-Fc by *N. caninum*

Forty-eight hours after infecting HFF cells with 3.10^6 of recombinant *N. caninum* tachyzoites, the culture medium was replaced with 2 mL of fresh medium. After 24 h of incubation, protozoa were removed by centrifugation, and scFv-Fc concentration was measured by ELISA. Concentrations obtained from the various supernatants used were approximately at 170 ng/mL. Enriched supernatant fractions were obtained from five T75 flasks (50 mL) after complete HFF cell lysis. Culture supernatants were concentrated to 500 μL with centrifugal ultrafiltration unit Amicon Ultra 15 (Merck Millipore) before injection into a Superdex 75 10/300 GL column (GE Healthcare Life Sciences, 17-5174-01) on an AKTA chromatography system. Proteins were eluted with PBS at a 0.5 mL/min flow rate and the fraction corresponding to the proteins at the expected size (110 kDa) was collected. Collected fraction was used for the following experiments with a concentration of scFv-Fc obtained at 10 μg of scFv-Fc/mL.

Quantification of the secreted scFv-Fc by recombinant *N. caninum* strain

Secreted scFv-Fc was quantified by sandwich ELISA.[73] Known concentrations of scFv-Fc from transfected CHO cells diluted in culture supernatant of Nc-1 were used as a standard. Briefly, *Peptostreptococcus* Protein L (PpL, GE Healthcare) in PBS (1 μg/mL) was coated overnight at 4°C into a 96-well flat-bottom plate (Maxisorp, Nunc). Coated wells were then blocked with PBS containing 4% bovine serum albumin (BSA, Sigma) for 2 h at 37°C. Samples and standard were added to the coated PpL and incubated for 2 h at 37°C. Microplates were incubated with horseradish peroxidase (HRP) conjugated goat anti-mouse IgG2a antibody (BD Pharmingen) followed by 3,3′,5,5′ tetramethylbenzidine (TMB) substrate (Sigma-Aldrich). The reaction was stopped with 1M H_2SO_4 and the absorbance was read at 450 nm.

SDS-PAGE and immunoblot

An anti-PD-L1 scFv-Fc produced by Chinese hamster ovary (CHO) cells was used as a reference. Indicated quantities of CHO-produced scFv-Fc, Nc-1-scFv-Fc culture supernatants and enriched supernatants were electrophoresed under non-reducing conditions on 10% acrylamide-bisacrylamide gel (SDS-PAGE) then transferred to nitrocellulose membrane, probed with anti-mouse IgG conjugated with HRP. Detection of signal was observed by chemiluminescent HRP substrate (SuperSignal West Pico PLUS Chemiluminescent Substrate, ThermoFisher Scientific).

For immunoblot with scFv from transfected CHO cells or culture supernatant of Nc-1-scFv, a similar procedure was applied, and nitrocellulose membrane was probed with primary antibodies rabbit anti-HA polyclonal antibody followed by anti-rabbit secondary antibody conjugated to HRP (Invitrogen).

Analysis of the secretion pathway

HFF cells in T75 culture flasks were infected with Nc-1-scFv-Fc tachyzoites. The number of extracellular tachyzoites present in the culture supernatant was determined by direct counting using a Malassez counting chamber under optical microscopy, ensuring accurate estimation for subsequent experiments. Infected cells were maintained at 15°C and scraped and passed through a 27-gauge needle to release intracellular tachyzoites. Tachyzoites were then washed, resuspended to a concentration of 2.10^7/mL in culture medium (10% FBS, 2% HEPES) and kept at 10°C until used. For ethanol-induced secretion, 1.10^6 tachyzoites in 50 μL culture medium was added to 50 μL of culture medium with 2% ethanol or without ethanol (control) prewarmed at 37°C in a 96-well plate and incubated at 37°C for 2 min. For temperature effect assay, tachyzoites were collected using the same protocol and incubated in culture medium at temperatures of 4°C–37°C for 30 min. For the kinetic of secretion, tachyzoites were collected using the same protocol and incubated at 37°C for various time points, giving an insight into the behavior of recombinant *N. caninum* strain under conditions corresponding to its intended therapeutic use. All secretion assays were arrested by placing the plates on ice for 5 min. Supernatants were collected after centrifugation at 800 × *g* for 10 min at 4°C and secreted scFv-Fc was measured by ELISA.

Binding of scFv-Fc anti-PD-L1 to PD-L1 expressing tumor cells and competition assay

MDA-MB-231 cells (5.10^4) were incubated for 30 min at 4°C with 100 μL of various concentrations of purified scFv-Fc from transfected CHO cells or with dilutions of culture supernatant from Nc-1-scFv-Fc. Cells were then washed, and binding of scFv-Fc was detected using APC-conjugated F(ab')2-goat anti-mouse IgG (H + L) (eBioscience) incubated for 30 min at 4°C. Finally, cells were analyzed by a MACSQuant10 flow cytometer (Miltenyi Biotec). For the binding of the secreted scFv-Fc to uninfected cells, MDA-MB-231 cells infected with different multiplicities of infection (MOIs) for 48 h and binding of scFv-Fc to infected and uninfected cells was analyzed using the same protocol. For competition assays, MDA-MB231 cells were blocked with increased concentrations of atezolizumab (Tecentriq Roche) for 30 min at 4°C. Then, cells were incubated in culture supernatant of Nc-1-scFv-Fc supernatant and binding of scFv-Fc was analyzed using the same protocol.

For fixation on B16F10 cells, similar protocol was applied with incubation of cells in PBS, culture supernatant of Nc-1-scFv-Fc or Nc-1-scFv, and stained with PpL-PE diluted at 10 for 30 min at 4°C.

For fixation of scFv-Fc purified from CHO cells or atezolizumab on MDA-MB-231 cells, similar protocol was applied and cells were stained with PpL-PE.

Neutralizing activity

B16F10 cells (1.10^4) were incubated with either culture supernatants from recombinant strain or controls for 45 min at 4°C. Then, cells were washed twice and incubated with biotinylated recombinant human PD-1 Fc Chimera (BioLegend) protein at 7 μg/mL in PBS containing 1% FBS for 45 min at 4°C. Cells were then washed twice and stained with FITC-Streptavidin (BD Pharmingen) for 30 min at 4°C. Finally, cells were analyzed by a MACSQuant10 flow cytometer (Miltenyi Biotec).

Co-culture of B16F10 tumor cells and B3Z CD8+ T cells

B3Z assay with B16F10 cells was performed as previously described.[19] Briefly, B3Z cells were co-cultured with B16F10 cells loaded with MHCI-restricted peptide SIINFEKL OVA 257–264 (ratio 1:1) in the presence of culture supernatant of Nc-1 or Nc-1-scFv-Fc (total volume of 100 μL/well). These co-cultures were incubated for 16 h at 37°C, and then 90 μL of supernatants were harvested and assessed for interleukin-2 (IL-2) secretion using an ELISA kit (Invitrogen) according to manufacturer's protocol.

Phagocytosis assays

Cell surface binding of scFv-Fc to RAW 264.7 cells was confirmed by flow cytometry using PpL coupled with phycoerythrin (PpL-PE, Sino Biological). Subsequently, mCherry/luc B16F10 cells were co-cultured in an ultra-low adherent 96-well plate (Corning) with RAW 264.7 macrophage cells at a ratio 1:1 in the presence of culture supernatant of Nc-1, Nc-1-scFv, Nc-1-scFv-Fc, or scFv-Fc from CHO (170 ng/mL) diluted in Nc-1 culture supernatant. Co-cultures were incubated for 5 h, then washed, stained with FITC-CD11b (Miltenyi Biotec), and analyzed with CytoFLEX Flow Cytometer (Beckman). The number of phagocytic macrophages was estimated by counting the number of double-positive events (mCherry+/FITC+).

Oncolytic activity and replication of *N. caninum* strains in B16F10 cells

B16F10 cells were seeded into p24-well at densities of 9.10^4 cells/well before infected with tachyzoites Nc-1 or Nc-1-scFv-Fc at MOIs of 0.156, 0.625, 1.25, 2.5, 5, or 10. After 72 h, cells were transferred into white flat-bottom p96-well (Costar). Medium was supplemented with 150 U/mL of D-luciferin (VivoGlo Luciferin, Promega) and the bioluminescence activity was measured with GloMax Luminometer (Promega). Cell viability was measured with respect to the control uninfected cells. For the replication assay, 2.10^5 B16F10 cells were seeded in T25 flasks for 6 h, before infection with Nc-1 or Nc-1-scFv-Fc at MOI 2. Twenty-four hours post infection (hpi), media was replaced with 4 mL fresh media. Cell culture media were harvested 48, 72, and 96 hpi and extracellular tachyzoites were counted by optical microscopy.

Analysis of antibody-dependent cellular cytotoxicity

A commercially available murine ADCC Reporter Bioassay was used according to manufacturer's instructions (Promega). Briefly, 1×10^4 MDA-MB231 cells/well (target cells) were seeded in a 96-well plate 24 h before the assay, then serial dilutions of culture supernatants of Nc-1 and Nc-1 scFv-Fc were added. ADCC bioassay effector cells

were added and after incubation for 6 h at 37 °C, the plates were kept on the bench for 15 min. Then 75 μL of Bio-Glo Luciferase reagent (Promega) was added in each well and kept at room temperature (RT) for 10 min. Luminescence activity was measured with GloMax Microplate Reader & Luminometer (Promega).

Immunolocalization by immunofluorescence

For subcellular localization of scFv-Fc in recombinant *N. caninum*, indirect immunofluorescence assays were performed on intracellular tachyzoites grown overnight in HFF cells on coverslip (Fisher Scientific) at MOI 1. Infected HFF monolayers were washed in PBS, fixed in PBS containing 4% PAF for 20 min at RT, then permeabilized using 0.2% Triton X-100 in PBS for 20 min and blocked with 3% FBS in PBS for 30 min. Cells were then incubated with rabbit anti-MIC3 serum diluted in PBS containing 1% FBS for 2 h at RT, washed and incubated for 1 h with secondary antibodies, Alexa 594 anti-rabbit IgG and Alexa Fluor 350 anti-mouse IgG (dilution 1:1000) (Thermo Fisher Scientific). After washes, slides were mounted using Immu-Mount (Thermo Scientific) and images were captured with Olympus IX73 fluorescent microscope using cellSens Dimension software.

For immunolocalization of the secreted scFv-Fc, IFN-γ-stimulated B16F10-K1 cells as well as rested (non-stimulated) B16F10-K1 cells plated on coverslips were infected with Nc-1-scFv-Fc at MOI 0.5. Finally, cells were washed in PBS, fixed in 4% PAF for 20 min at RT, then stained with APC-conjugated F(ab')2 anti-Mouse IgG (H + L) (eBioscience). After five washes, nuclei were stained using Hoechst and slides were mounted using Immu-Mount (Thermo Scientific). Cells were imaged with CSLM (Nikon AX, Nikon Europe B.V., Amsterdam, Netherlands) using a water-immersion Plan Apo VC 60×/1.2 objective lens and the NIS-Element AR software (version 5.42.06). Fiji software (version 1.54f, https://imagej.net/software/fiji/downloads) was used after for image visualization.[74]

Invasion assay

HFF cells in P24-wells were infected with 5.10^6 tachyzoites per well for 30 min or 3 h. After three washes, attached extracellular tachyzoites were fixed with 4% PAF and stained with anti-*N. caninum* (infection serum from rabbit) followed by Alexa fluor 594-conjugated goat anti-rabbit IgG at RT for 2 h. After permeabilization with 0.2% Triton X-100 in PBS for 20 min and incubation with PBS 3% FBS for 30 min, tachyzoites were stained overnight at 4°C with rabbit anti-*N. caninum* serum followed by Alexa fluor 594-conjugated goat anti-rabbit IgG at RT for 2 h. Cell nuclei were stained using Hoechst. Attached extracellular (double stained, yellow) and intracellular (green) tachyzoites were counted with EVOS M7000. Data were obtained by counting five fields at ×20 magnification.

Statistical analysis

All analysis was performed using GraphPad Prism 8.00 Software. Due to the non-normal distribution of the data, statistical significance was analyzed by the non-parametric Mann-Whitney and Kruskal-Wallis tests. Estimation of the Gaussian distribution of the data was performed using Shapiro-Wilk and D'Agostino-Pearson normality tests. All the data are displayed as medians and interquartile ranges. Statistical differences are indicated with asterisks: $^*p < 0.05$, $^{**}p < 0.01$.

DATA AVAILABILITY

All data generated or analyzed during this study are included in the main text or supplemental information. Further enquiries are directed to the corresponding author (A.d.T.), upon reasonable request.

ACKNOWLEDGMENTS

We thank Professor Emilie Allard-Vannier, Dr. Mehdi Khaled, Dr. Laurent Gros, Dr. Hans Acha-Orbea, and Dr. Nicolas Blanchard for providing the cell lines used in this study. We sincerely thank Dr. Florence Velge-Roussel for her expertise in flow cytometry and Dr. Julien Pichon for his contribution with microscopy study. We are also gracious for Jean-François Dubremetz and Diana Marcela Penarete-Vargas for providing, respectively, the anti-MIC3 of *Toxoplasma gondii* antibodies and serum of anti-*Neospora caninum*. This work was supported by the Région Centre-Val de Loire, the Laboratoire d'Excellence (LabEx) "MabImprove" (to C.R.), and the University of Tours, France.

AUTHOR CONTRIBUTIONS

Conceptualization, C.R., N.A., and A.d.T.; methodology, C.R., M.-N.M., S.G., N.M., N.A., and A.d.T.; investigation, C.R., M.A., M.-N.M., L.Lantier, F.B., L.Lajoie), C.D., N.M., and A.d.T.; formal analysis, C.R., M.A., M.-N.M., L.Lantier, N.M., N.A., and A.d.T.; writing – original draft preparation, C.R.; writing – review and editing, M.A., M.-N.M., I.D.-P., N.A., and A.d.-T.; supervision, M.-N.M., I.D.-P., N.A., and A.d.T. All authors have read and agreed to the published version of the manuscript.

DECLARATION OF INTERESTS

The authors declare no competing interests.

SUPPLEMENTAL INFORMATION

Supplemental information can be found online at https://doi.org/10.1016/j.omton.2025.200968.

REFERENCES

1. Zhang, Y., and Zhang, Z. (2020). The history and advances in cancer immunotherapy: understanding the characteristics of tumor-infiltrating immune cells and their therapeutic implications. Cell. Mol. Immunol. *17*, 807–821. https://doi.org/10.1038/s41423-020-0488-6.
2. Tan, S., Day, D., Nicholls, S.J., and Segelov, E. (2022). Immune Checkpoint Inhibitor Therapy in Oncology. JACC. CardioOncol. *4*, 579–597. https://doi.org/10.1016/j.jaccao.2022.09.004.
3. Twomey, J.D., and Zhang, B. (2021). Cancer Immunotherapy Update: FDA-Approved Checkpoint Inhibitors and Companion Diagnostics. AAPS J. *23*, 39. https://doi.org/10.1208/s12248-021-00574-0.
4. Jenkins, R.W., Barbie, D.A., and Flaherty, K.T. (2018). Mechanisms of resistance to immune checkpoint inhibitors. Br. J. Cancer *118*, 9–16. https://doi.org/10.1038/bjc.2017.434.
5. Tang, T., Huang, X., Zhang, G., Hong, Z., Bai, X., and Liang, T. (2021). Advantages of targeting the tumor immune microenvironment over blocking immune checkpoint in cancer immunotherapy. Signal Transduct. Target. Ther. *6*, 72. https://doi.org/10.1038/s41392-020-00449-4.
6. Abou Khouzam, R., Zaarour, R.F., Brodaczewska, K., Azakir, B., Venkatesh, G.H., Thiery, J., Terry, S., and Chouaib, S. (2022). The Effect of Hypoxia and Hypoxia-Associated Pathways in the Regulation of Antitumor Response: Friends or Foes? Front. Immunol. *13*, 828875. https://doi.org/10.3389/fimmu.2022.828875.
7. Liu, J., Bai, Y., Li, Y., Li, X., and Luo, K. (2024). Reprogramming the immunosuppressive tumor microenvironment through nanomedicine: an immunometabolism perspective. EBioMedicine *107*, 105301. https://doi.org/10.1016/j.ebiom.2024.105301.
8. Baxter, L.T., and Jain, R.K. (1989). Transport of fluid and macromolecules in tumors. I. Role of interstitial pressure and convection. Microvasc. Res. *37*, 77–104. https://doi.org/10.1016/0026-2862(89)90074-5.

9. Shalhout, S.Z., Miller, D.M., Emerick, K.S., and Kaufman, H.L. (2023). Therapy with oncolytic viruses: progress and challenges. Nat. Rev. Clin. Oncol. *20*, 160–177. https://doi.org/10.1038/s41571-022-00719-w.

10. Duong, M.T.Q., Qin, Y., You, S.H., and Min, J.J. (2019). Bacteria-cancer interactions: bacteria-based cancer therapy. Exp. Mol. Med. *51*, 1–15. https://doi.org/10.1038/s12276-019-0297-0.

11. Kleemann, J., Jäger, M., Valesky, E., Kippenberger, S., Kaufmann, R., and Meissner, M. (2021). Real-World Experience of Talimogene Laherparepvec (T-VEC) in Old and Oldest-Old Patients with Melanoma: A Retrospective Single Center Study. Cancer Manag. Res. *13*, 5699–5709. https://doi.org/10.2147/CMAR.S286917.

12. Lovatt, C., and Parker, A.L. (2023). Oncolytic Viruses and Immune Checkpoint Inhibitors: The “Hot” New Power Couple. Cancers *15*, 4178. https://doi.org/10.3390/cancers15164178.

13. Long, G.V., Dummer, R., Ribas, A., Puzanov, I., Michielin, O., VanderWalde, A., Andtbacka, R.H., Cebon, J., Fernandez, E., Malvehy, J., et al. (2015). A Phase I/III, multicenter, open-label trial of talimogene laherparepvec (T-VEC) in combination with pembrolizumab for the treatment of unresected, stage IIIb-IV melanoma (MASTERKEY-265). J. Immunother. Cancer *3* (*Suppl 2*), P181. https://doi.org/10.1186/2051-1426-3-S2-P181.

14. Puzanov, I., Milhem, M.M., Minor, D., Hamid, O., Li, A., Chen, L., Chastain, M., Gorski, K.S., Anderson, A., Chou, J., et al. (2016). Talimogene Laherparepvec in Combination With Ipilimumab in Previously Untreated, Unresectable Stage IIIB-IV Melanoma. J. Clin. Oncol. *34*, 2619–2626. https://doi.org/10.1200/JCO.2016.67.1529.

15. Ju, F., Luo, Y., Lin, C., Jia, X., Xu, Z., Tian, R., Lin, Y., Zhao, M., Chang, Y., Huang, X., et al. (2022). Oncolytic virus expressing PD-1 inhibitors activates a collaborative intratumoral immune response to control tumor and synergizes with CTLA-4 or TIM-3 blockade. J. Immunother. Cancer *10*, e004762. https://doi.org/10.1136/jitc-2022-004762.

16. Junqueira, C., Santos, L.I., Galvão-Filho, B., Teixeira, S.M., Rodrigues, F.G., DaRocha, W.D., Chiari, E., Jungbluth, A.A., Ritter, G., Gnjatic, S., et al. (2011). Trypanosoma cruzi as an effective cancer antigen delivery vector. Proc. Natl. Acad. Sci. USA *108*, 19695–19700. https://doi.org/10.1073/pnas.1110030108.

17. Chen, J., Liao, W., and Peng, H. (2022). Toxoplasma gondii infection possibly reverses host immunosuppression to restrain tumor growth. Front. Cell. Infect. Microbiol. *12*, 959300. https://doi.org/10.3389/fcimb.2022.959300.

18. Chen, X., Qin, L., Hu, W., and Adah, D. (2021). The mechanisms of action of Plasmodium infection against cancer. Cell Commun. Signal. *19*, 74. https://doi.org/10.1186/s12964-021-00748-5.

19. Aljieli, M., Rivière, C., Lantier, L., Moiré, N., Lakhrif, Z., Boussemart, A.F., Cnudde, T., Lajoie, L., Aubrey, N., Ahmed, E.M., et al. (2024). Specific Cell Targeting by Toxoplasma gondii Displaying Functional Single-Chain Variable Fragment as a Novel Strategy; A Proof of Principle. Cells *13*, 975. https://doi.org/10.3390/cells13110975.

20. Zhu, Y.C., Elsheikha, H.M., Wang, J.H., Fang, S., He, J.J., Zhu, X.Q., and Chen, J. (2021). Synergy between Toxoplasma gondii type I ΔGRA17 immunotherapy and PD-L1 checkpoint inhibition triggers the regression of targeted and distal tumors. J. Immunother. Cancer *9*, e002970. https://doi.org/10.1136/jitc-2021-002970.

21. Bahwal, S.A., Chen, J.J., E, L., Hao, T., Chen, J., Carruthers, V.B., Lai, J., and Zhou, X. (2022). Attenuated Toxoplasma gondii enhances the antitumor efficacy of anti-PD1 antibody by altering the tumor microenvironment in a pancreatic cancer mouse model. J. Cancer Res. Clin. Oncol. *148*, 2743–2757. https://doi.org/10.1007/s00432-022-04036-8.

22. Ellis, J., Luton, K., Baverstock, P.R., Brindley, P.J., Nimmo, K.A., and Johnson, A.M. (1994). The phylogeny of Neospora caninum. Mol. Biochem. Parasitol. *64*, 303–311. https://doi.org/10.1016/0166-6851(94)00033-6.

23. Lantier, L., Poupée-Beaugé, A., di Tommaso, A., Ducournau, C., Epardaud, M., Lakhrif, Z., Germon, S., Debierre-Grockiego, F., Mévélec, M.N., Battistoni, A., et al. (2020). Neospora caninum: a new class of biopharmaceuticals in the therapeutic arsenal against cancer. J. Immunother. Cancer *8*, e001242. https://doi.org/10.1136/jitc-2020-001242.

24. Battistoni, A., Lantier, L., di Tommaso, A., Ducournau, C., Lajoie, L., Samimi, M., Coënon, L., Rivière, C., Epardaud, M., Hertereau, L., et al. (2023). Nasal administration of recombinant Neospora caninum secreting IL-15/IL-15Rα inhibits metastatic melanoma development in lung. J. Immunother. Cancer *11*, e006683. https://doi.org/10.1136/jitc-2023-006683.

25. Li, X., Qi, M., He, K., Liu, H., Yan, W., Zhao, L., Jia, Y., He, L., Lv, C., Zhang, M., et al. (2022). Neospora caninum inhibits tumor development by activating the immune response and destroying tumor cells in a B16F10 melanoma model. Parasites Vectors *15*, 332. https://doi.org/10.1186/s13071-022-05456-8.

26. Dubois, D.J., and Soldati-Favre, D. (2019). Biogenesis and secretion of micronemes in Toxoplasma gondii. Cell. Microbiol. *21*, e13018. Erratum in: Cell Microbiol. 2019 Oct;21(10):e13105. doi: 10.1111/cmi.13105. https://doi.org/10.1111/cmi.13018.

27. Striepen, B., He, C.Y., Matrajt, M., Soldati, D., and Roos, D.S. (1998). Expression, selection, and organellar targeting of the green fluorescent protein in *Toxoplasma gondii*. Mol. Biochem. Parasitol. *92*, 325–338. https://doi.org/10.1016/S0166-6851(98)00011-5.

28. Tagoe, D.N.A., Drozda, A.A., Falco, J.A., Bechtel, T.J., Weerapana, E., and Gubbels, M.J. (2021). Ferlins and TgDOC2 in *Toxoplasma* Microneme, Rhoptry and Dense Granule Secretion. Life *11*, 217. https://doi.org/10.3390/life11030217.

29. Carruthers, V.B., Moreno, S.N., and Sibley, L.D. (1999). Ethanol and acetaldehyde elevate intracellular [Ca2+] and stimulate microneme discharge in Toxoplasma gondii. Biochem. J. *342*, 379–386.

30. Carruthers, V.B., Giddings, O.K., and Sibley, L.D. (1999). Secretion of micronemal proteins is associated with toxoplasma invasion of host cells. Cell. Microbiol. *1*, 225–235. https://doi.org/10.1046/j.1462-5822.1999.00023.x.

31. Carruthers, V.B., and Sibley, L.D. (1997). Sequential protein secretion from three distinct organelles of Toxoplasma gondii accompanies invasion of human fibroblasts. Eur. J. Cell Biol. *73*, 114–123.

32. Carruthers, V.B., and Tomley, F.M. (2008). Microneme Proteins in Apicomplexans. In Molecular Mechanisms of Parasite Invasion: Subcellular Biochemistry, B.A. Burleigh and D. Soldati-Favre, eds. (Springer), pp. 33–45. https://doi.org/10.1007/978-0-387-78267-6_2.

33. Cowman, A.F., Tonkin, C.J., Tham, W.H., and Duraisingh, M.T. (2017). The Molecular Basis of Erythrocyte Invasion by Malaria Parasites. Cell Host Microbe *22*, 232–245. https://doi.org/10.1016/j.chom.2017.07.003.

34. Paing, M.M., and Tolia, N.H. (2014). Multimeric Assembly of Host-Pathogen Adhesion Complexes Involved in Apicomplexan Invasion. PLoS Pathog. *10*, e1004120. https://doi.org/10.1371/journal.ppat.1004120.

35. Gogesch, P., Dudek, S., van Zandbergen, G., Waibler, Z., and Anzaghe, M. (2021). The Role of Fc Receptors on the Effectiveness of Therapeutic Monoclonal Antibodies. Int. J. Mol. Sci. *22*, 8947. https://doi.org/10.3390/ijms22168947.

36. Ramesh, A., Kumar, S., Nandi, D., and Kulkarni, A. (2019). CSF1R- and SHP2-Inhibitor-Loaded Nanoparticles Enhance Cytotoxic Activity and Phagocytosis in Tumor-Associated Macrophages. Adv. Mater. *31*, e1904364. https://doi.org/10.1002/adma.201904364.

37. Ngwa, W., Irabor, O.C., Schoenfeld, J.D., Hesser, J., Demaria, S., and Formenti, S.C. (2018). Using immunotherapy to boost the abscopal effect. Nat. Rev. Cancer *18*, 313–322. https://doi.org/10.1038/nrc.2018.6.

38. Das, S., and Johnson, D.B. (2019). Immune-related adverse events and anti-tumor efficacy of immune checkpoint inhibitors. J. Immunother. Cancer *7*, 306. https://doi.org/10.1186/s40425-019-0805-8.

39. Schonfeld, S.J., Tucker, M.A., Engels, E.A., Dores, G.M., Sampson, J.N., Shiels, M.S., Chanock, S.J., and Morton, L.M. (2022). Immune-Related Adverse Events After Immune Checkpoint Inhibitors for Melanoma Among Older Adults. JAMA Netw. Open *5*, e223461. https://doi.org/10.1001/jamanetworkopen.2022.3461.

40. Huang, A.C., and Zappasodi, R. (2022). A decade of checkpoint blockade immunotherapy in melanoma: understanding the molecular basis for immune sensitivity and resistance. Nat. Immunol. *23*, 660–670. https://doi.org/10.1038/s41590-022-01141-1.

41. Topalian, S.L., Hodi, F.S., Brahmer, J.R., Gettinger, S.N., Smith, D.C., McDermott, D.F., Powderly, J.D., Carvajal, R.D., Sosman, J.A., Atkins, M.B., et al. (2012). Safety, Activity, and Immune Correlates of Anti–PD-1 Antibody in Cancer. N. Engl. J. Med. *366*, 2443–2454. https://doi.org/10.1056/NEJMoa1200690.

42. Tumeh, P.C., Harview, C.L., Yearley, J.H., Shintaku, I.P., Taylor, E.J.M., Robert, L., Chmielowski, B., Spasic, M., Henry, G., Ciobanu, V., et al. (2014). PD-1 blockade

induces responses by inhibiting adaptive immune resistance. Nature *515*, 568–571. https://doi.org/10.1038/nature13954.

43. Schultz, L., and Gardner, R. (2019). Mechanisms of and approaches to overcoming resistance to immunotherapy. Hematol Am Soc Hematol Educ Program *2019*, 226–232. https://doi.org/10.1182/hematology.2019000018.

44. Sivanandam, V., LaRocca, C.J., Chen, N.G., Fong, Y., and Warner, S.G. (2019). Oncolytic Viruses and Immune Checkpoint Inhibition: The Best of Both Worlds. Mol. Ther. Oncolytics *13*, 93–106. https://doi.org/10.1016/j.omto.2019.04.003.

45. Haddad, A.F., Young, J.S., and Aghi, M.K. (2021). Using viral vectors to deliver local immunotherapy to glioblastoma. Neurosurg. Focus *50*, E4. https://doi.org/10.3171/2020.11.FOCUS20859.

46. Bartee, M.Y., Dunlap, K.M., and Bartee, E. (2017). Tumor-localized secretion of soluble PD1 enhances oncolytic virotherapy. Cancer Res. *77*, 2952–2963. https://doi.org/10.1158/0008-5472.CAN-16-1638.

47. La Flamme, A.C., Buckner, F.S., Swindle, J., Ajioka, J., and Van Voorhis, W.C. (1996). *Trypanosoma cruzi:*Expression of Interleukin-2 Utilizing both Supercoiled Plasmids and Linear DNAs. Exp. Parasitol. *83*, 159–163. https://doi.org/10.1006/expr.1996.0061.

48. La Flamme, A.C., Buckner, F.S., Swindle, J., Ajioka, J., and Van Voorhis, W.C. (1995). Expression of mammalian cytokines by *Trypanosoma cruzi* indicates unique signal sequence requirements and processing. Mol. Biochem. Parasitol. *75*, 25–31. https://doi.org/10.1016/0166-6851(95)02506-5.

49. McFadden, D.C., Seeber, F., and Boothroyd, J.C. (1997). Use of Toxoplasma gondii expressing beta-galactosidase for colorimetric assessment of drug activity in vitro. Antimicrob. Agents Chemother. *41*, 1849–1853.

50. Nishikawa, Y., Xuenan, X., Makala, L., Vielemeyer, O., Joiner, K.A., and Nagasawa, H. (2003). Characterisation of *Toxoplasma gondii* engineered to express mouse interferon-gamma. Int. J. Parasitol. *33*, 1525–1535. https://doi.org/10.1016/S0020-7519(03)00204-2.

51. Unverdorben, F., Richter, F., Hutt, M., Seifert, O., Malinge, P., Fischer, N., and Kontermann, R.E. (2016). Pharmacokinetic properties of IgG and various Fc fusion proteins in mice. mAbs *8*, 120–128. https://doi.org/10.1080/19420862.2015.1113360.

52. Jørgensen, M.L., Friis, N.A., Just, J., Madsen, P., Petersen, S.V., and Kristensen, P. (2014). Expression of single-chain variable fragments fused with the Fc-region of rabbit IgG in Leishmania tarentolae. Microb. Cell Fact. *13*, 9. https://doi.org/10.1186/1475-2859-13-9.

53. Just, J., Lykkemark, S., Nielsen, C.H., Roshenas, A.R., Drasbek, K.R., Petersen, S.V., Bek, T., and Kristensen, P. (2017). Pericyte modulation by a functional antibody obtained by a novel single-cell selection strategy. Microcirculation *24*, e12365. https://doi.org/10.1111/micc.12365.

54. Brydges, S.D., Harper, J.M., Parussini, F., Coppens, I., and Carruthers, V.B. (2008). A transient forward targeting element for microneme regulated secretion in Toxoplasma gondii. Biol. Cell *100*, 253–264. https://doi.org/10.1042/BC20070076.

55. Naguleswaran, A., Cannas, A., Keller, N., Vonlaufen, N., Björkman, C., and Hemphill, A. (2002). Vero cell surface proteoglycan interaction with the microneme protein NcMIC3 mediates adhesion of *Neospora caninum* tachyzoites to host cells unlike that in *Toxoplasma gondii*. Int. J. Parasitol. *32*, 695–704. https://doi.org/10.1016/S0020-7519(02)00014-0.

56. Desbois, M., Le Vu, P., Coutzac, C., Marcheteau, E., Béal, C., Terme, M., Gey, A., Morisseau, S., Teppaz, G., Boselli, L., et al. (2016). IL-15 Trans-Signaling with the Superagonist RLI Promotes Effector/Memory CD8+ T Cell Responses and Enhances Antitumor Activity of PD-1 Antagonists. J. Immunol. *197*, 168–178. https://doi.org/10.4049/jimmunol.1600019.

57. Ng, S.S.M., Nagy, B.A., Jensen, S.M., Hu, X., Alicea, C., Fox, B.A., Felber, B.K., Bergamaschi, C., and Pavlakis, G.N. (2017). Heterodimeric IL-15 Treatment Enhances Tumor Infiltration, Persistence and Effector Functions of Adoptively Transferred Tumor-specific T Cells in the Absence of Lymphodepletion. Clin. Cancer Res. *23*, 2817–2830. https://doi.org/10.1158/1078-0432.CCR-16-1808.

58. Deng, R., Bumbaca, D., Pastuskovas, C.V., Boswell, C.A., West, D., Cowan, K.J., Chiu, H., McBride, J., Johnson, C., Xin, Y., et al. (2016). Preclinical pharmacokinetics, pharmacodynamics, tissue distribution, and tumor penetration of anti-PD-L1 monoclonal antibody, an immune checkpoint inhibitor. mAbs *8*, 593–603. https://doi.org/10.1080/19420862.2015.1136043.

59. Marabelle, A., Andtbacka, R., Harrington, K., Melero, I., Leidner, R., de Baere, T., Robert, C., Ascierto, P.A., Baurain, J.F., Imperiale, M., et al. (2018). Starting the fight in the tumor: expert recommendations for the development of human intratumoral immunotherapy (HIT-IT). Ann. Oncol. *29*, 2163–2174. https://doi.org/10.1093/annonc/mdy423.

60. Liu, H.C., Viswanath, D.I., Pesaresi, F., Xu, Y., Zhang, L., Di Trani, N., Paez-Mayorga, J., Hernandez, N., Wang, Y., Erm, D.R., et al. (2021). Potentiating Antitumor Efficacy Through Radiation and Sustained Intratumoral Delivery of Anti-CD40 and Anti-PDL1. Int. J. Radiat. Oncol. Biol. Phys. *110*, 492–506. https://doi.org/10.1016/j.ijrobp.2020.07.2326.

61. Sagiv-Barfi, I., Czerwinski, D.K., Levy, S., Alam, I.S., Mayer, A.T., Gambhir, S.S., and Levy, R. (2018). Eradication of spontaneous malignancy by local immunotherapy. Sci. Transl. Med. *10*, eaan4488. https://doi.org/10.1126/scitranslmed.aan4488.

62. Hossler, P., Khattak, S.F., and Li, Z.J. (2009). Optimal and consistent protein glycosylation in mammalian cell culture. Glycobiology *19*, 936–949. https://doi.org/10.1093/glycob/cwp079.

63. Jefferis, R. (2021). Recombinant Proteins and Monoclonal Antibodies. In Advances in Glycobiotechnology, E. Rapp and U. Reichl, eds. (Springer International Publishing), pp. 281–318. https://doi.org/10.1007/10_2017_32.

64. Luk, F.C.Y., Johnson, T.M., and Beckers, C.J. (2008). N-linked glycosylation of proteins in the protozoan parasite Toxoplasma gondii. Mol. Biochem. Parasitol. *157*, 169–178. https://doi.org/10.1016/j.molbiopara.2007.10.012.

65. Samuelson, J., and Robbins, P.W. (2015). Effects of N-glycan precursor length diversity on quality control of protein folding and on protein glycosylation. Semin. Cell Dev. Biol. *41*, 121–128. https://doi.org/10.1016/j.semcdb.2014.11.008.

66. Cohen Saban, N., Yalin, A., Landsberger, T., Salomon, R., Alva, A., Feferman, T., Amit, I., and Dahan, R. (2023). Fc glycoengineering of a PD-L1 antibody harnesses Fcγ receptors for increased antitumor efficacy. Sci. Immunol. *8*, eadd8005. https://doi.org/10.1126/sciimmunol.add8005.

67. Dahan, R., Sega, E., Engelhardt, J., Selby, M., Korman, A.J., and Ravetch, J.V. (2015). FcγRs Modulate the Anti-tumor Activity of Antibodies Targeting the PD-1/PD-L1 Axis. Cancer Cell *28*, 285–295. https://doi.org/10.1016/j.ccell.2015.08.004.

68. Tranas, J., Heinzen, R.A., Weiss, L.M., and McAllister, M.M. (1999). Serological evidence of human infection with the protozoan Neospora caninum. Clin. Diagn. Lab. Immunol. *6*, 765–767. https://doi.org/10.1128/CDLI.6.5.765-767.1999.

69. Lobato, J., Silva, D.A.O., Mineo, T.W.P., Amaral, J.D.H.F., Segundo, G.R.S., Costa-Cruz, J.M., Ferreira, M.S., Borges, A.S., and Mineo, J.R. (2006). Detection of immunoglobulin G antibodies to Neospora caninum in humans: high seropositivity rates in patients who are infected by human immunodeficiency virus or have neurological disorders. Clin. Vaccine Immunol. *13*, 84–89. https://doi.org/10.1128/CVI.13.1.84-89.2006.

70. Guerriaud, M., Poupet, C., Lakhrif, Z., Kohli, E., and Moiré, N. (2024). Are genetically modified protozoa eligible for ATMP status? Concerning the legal categorization of an oncolytic protozoan drug candidate. Gene Ther. *31*, 295–303. https://doi.org/10.1038/s41434-024-00445-1.

71. Penarete-Vargas, D.M., Mévélec, M.N., Dion, S., Sèche, E., Dimier-Poisson, I., and Fandeur, T. (2010). Protection against Lethal Neospora caninum Infection in Mice Induced by Heterologous Vaccination with a mic1 mic3 Knockout Toxoplasma gondii Strain. Infect. Immun. *78*, 651–660. https://doi.org/10.1128/IAI.00703-09.

72. Jolivet, L., Ait Mohamed Amar, I., Horiot, C., Boursin, F., Colas, C., Letast, S., Denevault-Sabourin, C., Allard-Vannier, E., Joubert, N., and Aubrey, N. (2022). Intra-Domain Cysteines (IDC), a New Strategy for the Development of Original Antibody Fragment–Drug Conjugates (FDCs). Pharmaceutics *14*, 1524. https://doi.org/10.3390/pharmaceutics14081524.

73. Lakhrif, Z., Pugnière, M., Henriquet, C., di Tommaso, A., Dimier-Poisson, I., Billiald, P., Juste, M.O., and Aubrey, N. (2015). A method to confer Protein L binding ability to any antibody fragment. mAbs *8*, 379–388. https://doi.org/10.1080/19420862.2015.1116657.

74. Schindelin, J., Arganda-Carreras, I., Frise, E., Kaynig, V., Longair, M., Pietzsch, T., Preibisch, S., Rueden, C., Saalfeld, S., Schmid, B., et al. (2012). Fiji: an open-source platform for biological-image analysis. Nat. Methods *9*, 676–682. https://doi.org/10.1038/nmeth.2019.

OMTON, Volume *33*

Supplemental information

Neospora caninum as delivery vehicle for anti-PD-L1 scFv-Fc: A novel approach for cancer immunotherapy

Clément Riviere, Muna Aljieli, Marie-Noëlle Mévélec, Louis Lantier, Fanny Boursin, Laurie Lajoie, Céline Ducournau, Stéphanie Germon, Nathalie Moiré, Isabelle Dimier-Poisson, Nicolas Aubrey, and Anne di Tommaso

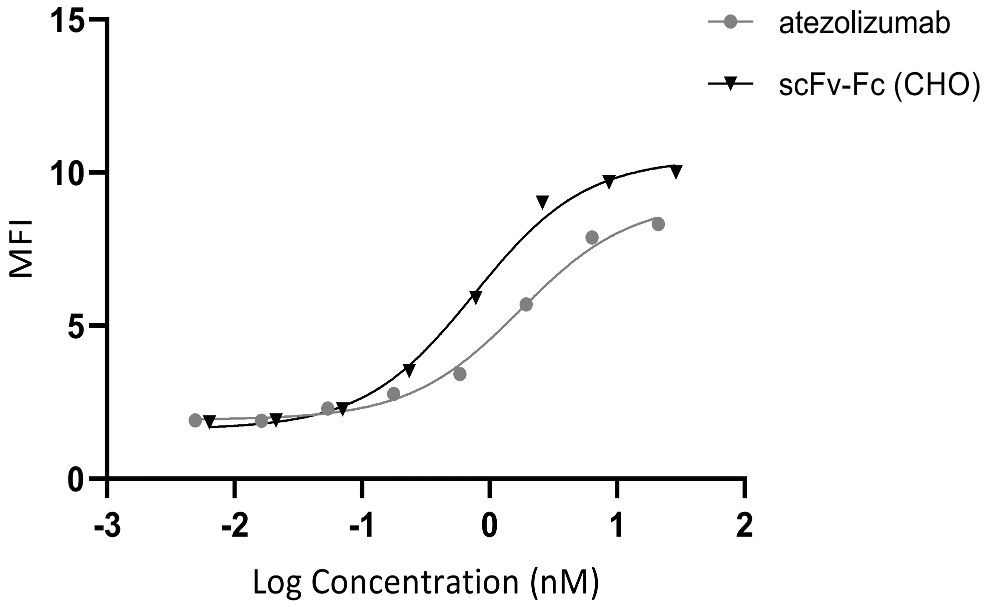

Figure S1: Anti-PD-L1 in scFv-Fc format binds to PD-L1+ tumor cells. Human tumor MDA-MB-231 cells were incubated with various concentrations of scFv-Fc purified from transfected CHO cells (scFv-Fc (CHO)) or atezolizumab (atezolizumab). MFI: Mean Fluorescent Intensity.

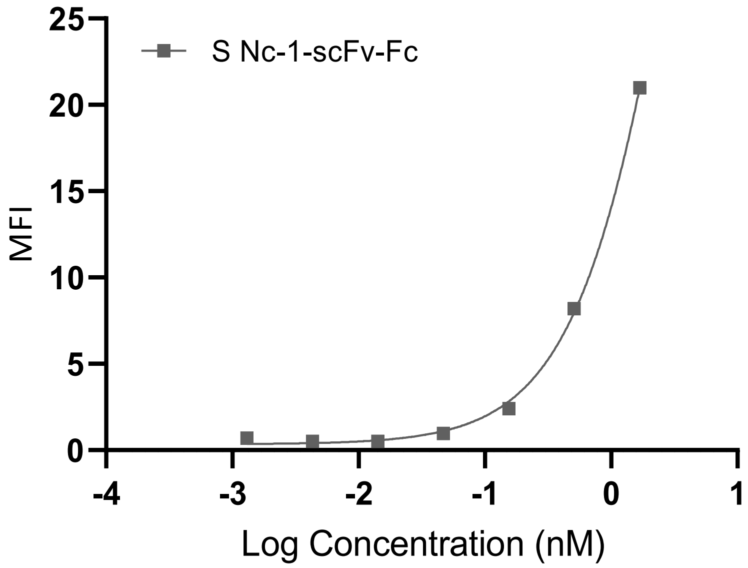

Figure S2: scFv-Fc produced by Nc-1-scFv-Fc binds to murine tumor cells. Mouse tumor cells B16F10 were incubated with various concentrations of secreted scFv-Fc corresponding to several dilutions culture supernatant of Nc-1-scFv-Fc (S Nc-1-scFv-Fc). MFI: Mean Fluorescent Intensity.

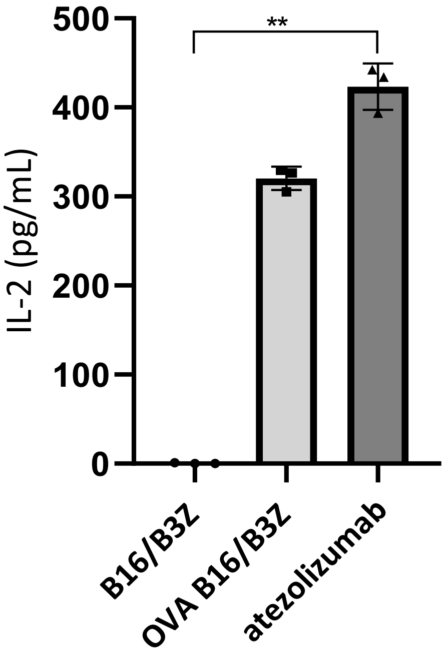

Figure S3: PD-1/PD-L1 axis limits secretion of IL-2 by B3Z cells. B16F10 cells overexpressing PD-L1 were pulsed with OVA peptide and then co-incubated with B3Z T cells (OVA B16/B3Z), in culture medium supplemented with atezolizumab (atezolizumab). As control, B16F10 cells without preincubation with OVA peptide (B16/B3Z) were co-incubated with B3Z T cells in culture medium. IL-2 production was measured by ELISA. Data are presented as mean values (n=3) and significant differences are indicated as **p < .01

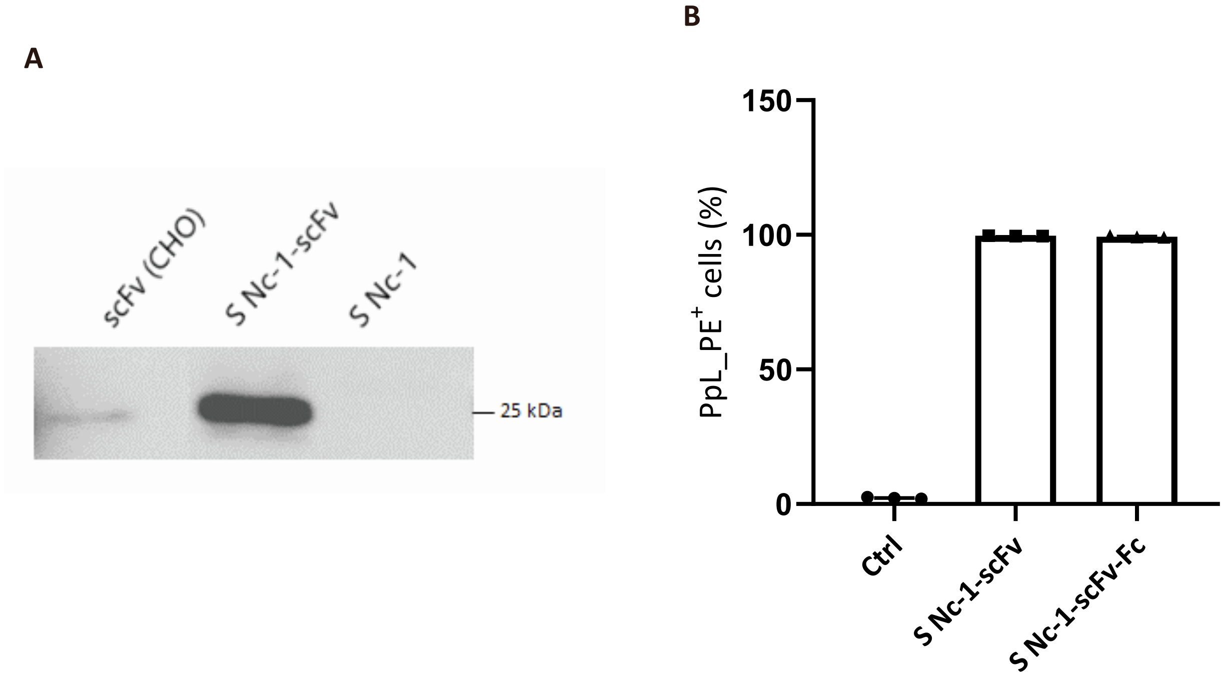

Figure S4: scFv from recombinant *Neospora caninum* binds to tumor cells. (A) Western blot was performed with culture supernatant of Nc-1-scFv (S Nc-1-scFv), culture supernatant of wild type strain (S Nc-1) and purified anti-PD-L1 scFv from CHO (scFv (CHO)). (B) Mouse tumor cells B16F10 were incubated with culture media of B16F10 (Ctrl), culture supernatant of Nc-1-scFv-Fc (S Nc-1-scFv-Fc) or culture supernatant of recombinant Nc-1-scFv (S Nc-1-scFv). Cells were analyzed by flow cytometry.

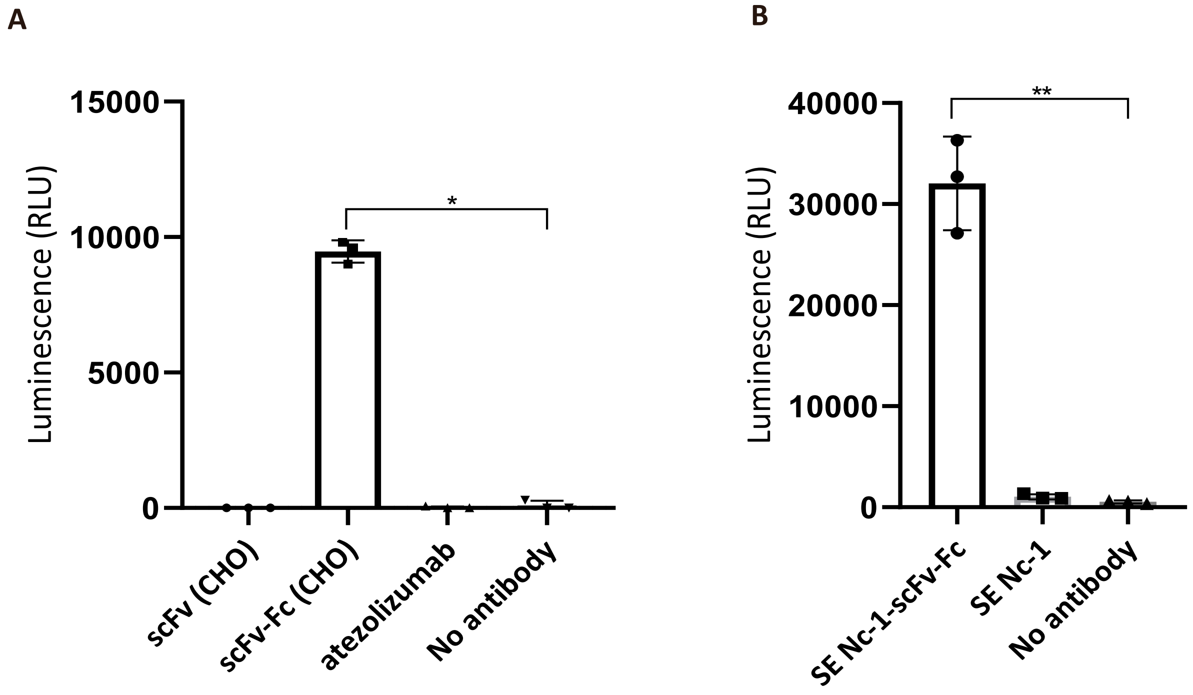

Figure S5: In vitro ADCC assay for antibody fragments produced by recombinant *N. caninum* or CHO. (A) ADCC Reporter Bioassay was made on MDA-MB-231. Target MDA-MB-231 cells were incubated with PBS (No antibody),atezolizumab (atezolizumab), or anti-PD-L1 in scFv format from CHO (scFv (CHO)) or anti-PD-L1 in scFv-Fc format from CHO (scFv-fc (CHO)) and followed, for each condition, by addition of effector cells. Luminescence was measured in relative light units. (B) In vitro ADCC assay with PBS (No antibody), enriched culture supernatant of wild type strain (SE Nc-1), enriched culture supernatant of Nc-1-scFv-Fc (SE Nc-1-scFv-Fc). Data are presented as means values (n=3) and significant differences are indicated as **p* < .05; ***p* < .01.

A

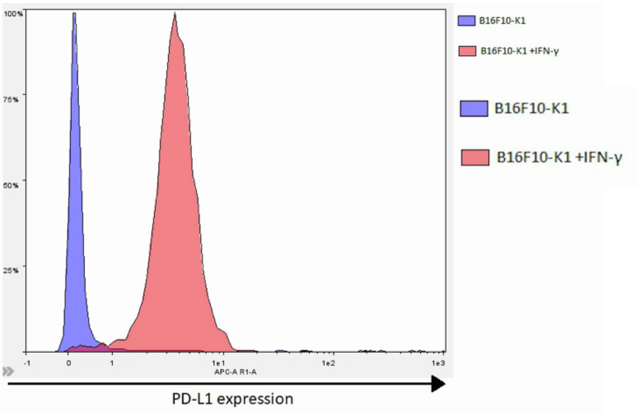

B

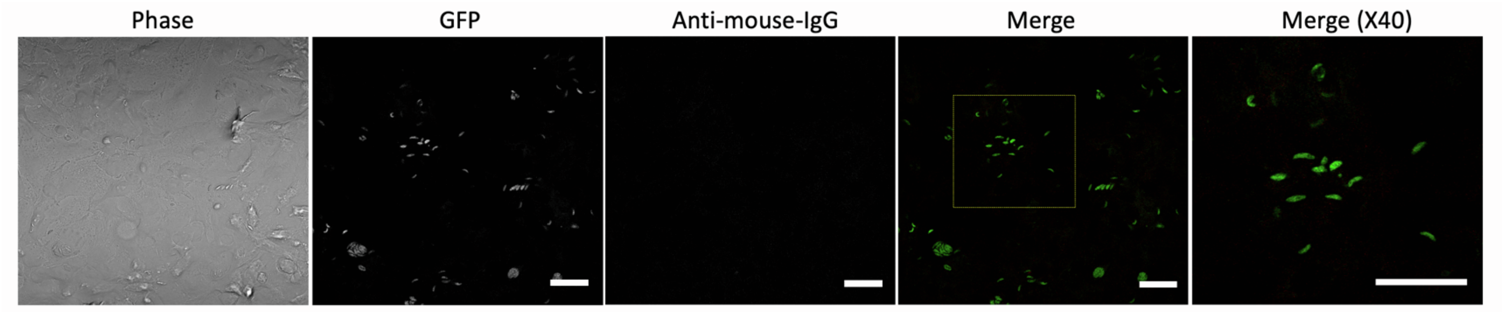

Figure S6: Binding of secreted scFv-Fc on infected or uninfected rested (non-stimulated) B16F10-K1. (A) Surface display level of PD-L1 on IFN-γ-stimulated B16F10-K1 (B16F10-K1 + IFN-γ) and non-stimulated B16F10-K1 (B16F10-K1) cells. Cells were stained with APC-conjugated mouse anti-PD-L1 before flow cytometry analysis. Results are represented as flow cytometer histograms. Profile of cells stained with anti-PD-L1 are indicated in blue area (non-stimulated B16F10-K1) and red area (IFN-γ-stimulated B16F10-K1). (B) Binding of scFv-Fc on uninfected or infected rested (non-stimulated) B16F10-K1 cells by immunofluorescence microscopy. Non-stimulated B16F10-K1 cells were incubated with Nc-1-scFv-Fc. Secreted scFv-Fc was detected with APC-conjugated anti-murine IgG (red) and individual extracellular and intracellular Nc-1-scFv-Fc were determined according to GFP expression (green). Representative results from 1 of 3 independent experiments are shown. Scale bar= 30 µm.
